# Abnormal mechanical load aggravates subchondral bone remodeling and uneven tibial plateau settlement in knee osteoarthritis via activation of osteoblast Piezo1-Ca²⁺-JAK2/STAT3 signaling

**DOI:** 10.7150/ijbs.124507

**Published:** 2026-01-15

**Authors:** Handi Li, Shuo Zhang, Chen Feng, Fangyan Cheng, Yuanyuan Han, Mengxue Wang, Shuai Zhou, Wenbo Shao, Wenzhong Chen, Jingguo Chen, Kai Liu, Yingze Zhang, Juan Wang

**Affiliations:** 1 Department of Orthopaedic Surgery, The Third Hospital of Hebei Medical University, Shijiazhuang, China.; 2 Hebei Medical University, Shijiazhuang, China.; 3 School of Medicine, Nankai University, Tianjin, China.

**Keywords:** knee osteoarthritis, uneven tibial plateau settlement, subchondral bone, osteoblast, Piezo1, JAK2/STAT3 signaling

## Abstract

Mechanical overload is closely associated with the theory of uneven tibial plateau settlement in knee osteoarthritis (KOA). Excessive mechanical stress leads to abnormal force distribution within the subchondral bone, eventually inducing medial tibial plateau collapse. This process disrupts local biomechanical homeostasis and triggers aberrant bone remodeling. However, the precise molecular basis of subchondral bone remodeling and structural changes in the knee is still not fully understood. In this work, we employed a mouse model of KOA with osteoblast-specific Piezo1 deletion, together with *in vitro* loading experiments, to demonstrate that mechanical overload activates Piezo1, promotes Ca²⁺ influx, and drives osteoblast differentiation, thereby contributing to subchondral bone sclerosis. Mechanistic investigations revealed that inhibition of the Piezo1-JAK2/STAT3 signaling axis alleviated abnormal osteoblast activation and significantly ameliorated subchondral bone sclerosis and cartilage degeneration. Moreover, deletion of JAK2 in osteoblasts further confirmed that blockade of this pathway mitigates KOA progression *in vivo*. Collectively, our findings identify the Piezo1-Ca²⁺-JAK2/STAT3 axis as a key mediator of osteoblast mechanotransduction under pathological loading and a potential therapeutic target for mechanical overload-associated KOA.

## Introduction

Knee osteoarthritis (KOA) is a major degenerative joint disorder with a high rate of disability, affecting more than 655 million people worldwide, and the number of patients in China alone is projected to exceed 400 million by 2030 1, 2. Most individuals with OA eventually develop varus knee deformity, which further aggravates functional impairment. The resulting long-term disability and medical burden pose a serious public health concern 3. From a biomechanical perspective, the knee joint exhibits inherent asymmetry: the medial tibial plateau bears nearly two-thirds of the load, while the lateral compartment, partially buffered by the fibula, supports only one-third 4-6. Biomechanical evidence suggests that the anteromedial tibial plateau of KOA patients experiences heightened stress concentrations, increasing the likelihood of structural instability and rendering the medial compartment particularly vulnerable to pathological changes under chronic overload 7. Drawing on the engineering concept of uneven foundation settlement and Wolff's law, we previously proposed the theory of “uneven tibial plateau settlement,” in which sustained medial overload drives microfracture accumulation and maladaptive bone remodeling 8-10. Such maladaptive remodeling initiates a vicious cycle of overload accumulation, plateau collapse, varus deformity, and mechanical imbalance that collectively accelerate KOA progression. While surgical approaches such as high tibial osteotomy (HTO) can alleviate pain and improve alignment, they are accompanied by surgical trauma, bone healing requirements, and potential perioperative risks 11-13. Thus, there is a pressing need to develop non-surgical strategies capable of interrupting this pathological process and effectively controlling disease progression.

Accumulating evidence indicates that abnormal mechanical loading is an important contributor to KOA progression 14, 15. KOA is increasingly viewed as a disease involving the entire joint, with dynamic crosstalk among cartilage, subchondral bone, and synovium 16-18. Subchondral bone is especially important in converting mechanical stimuli into biological responses and buffering excessive load, forming a bidirectional regulatory unit with the overlying cartilage 19-21. Clinical studies have demonstrated the association between structural alterations in subchondral bone, such as decreased modulus and trabecular disorganization, and excessive mechanical load-induced early cartilage degeneration 22. However, the mechanistic link between altered joint loading and the resulting tibial plateau settlement remains unclear.

Subchondral bone is continuously remodeled in adaptation to alterations in the mechanical milieu, with osteoblasts, osteocytes, and osteoclasts jointly contributing to its structural and functional integrity 23, 24. Among these, osteoblasts represent one of the mechanosensitive cell populations that translate mechanical cues into osteogenic responses 25, 26. Piezo1, a recently discovered mechanosensitive ion channel, forms a trimeric transmembrane structure that undergoes conformational changes in response to membrane tension and matrix deformation 27. These changes trigger rapid calcium influx into the cell. In osteoblasts, Piezo1-mediated Ca²⁺ signaling contributes to osteogenic differentiation and matrix mineralization 28-30. Dysregulation of Piezo1 expression or activity may impair bone homeostasis and has been associated with skeletal abnormalities, including osteoporosis and developmental bone defects 31, 32. Nevertheless, how Piezo1 contributes to load-driven subchondral bone remodeling in KOA remains poorly defined.

JAK2/STAT3 signaling is a canonical pathway that regulates cell survival, proliferation, and differentiation in many tissues, including bone 33. In the skeletal system, JAK2/STAT3 activity has been implicated in bone formation and remodeling 34. Aberrant activation of JAK2/STAT3 has also been reported in osteoarthritic joints, particularly in relation to inflammatory and catabolic changes in cartilage and synovium 35. However, its role in subchondral bone under abnormal mechanical loading remains poorly understood. In particular, whether Piezo1-mediated Ca²⁺ influx engages JAK2/STAT3 signaling in this context, and how such crosstalk contributes to subchondral bone sclerosis and uneven tibial plateau settlement in KOA, remains unclear.

Therefore, this study aimed to investigate the role of Piezo1 in osteoblast mechanotransduction under mechanical overload, to determine whether JAK2/STAT3 signaling acts downstream of Piezo1 activation, and to clarify how this axis influences subchondral bone remodeling and uneven tibial plateau settlement during KOA progression.

## Materials and Methods

### Ethics statement

Animal experiments were approved by the Animal Ethics Committee of Hebei Medical University (Approval No. IACUC-Hebmu-2023072). All experimental procedures, including surgical operations, were strictly conducted in compliance with the regulations of the Animal Ethics Committee.

### Human samples

This study was approved by the Ethics Committee of the Third Hospital of Hebei Medical University (Approval No. 2021-056-1) and conducted in accordance with the Declaration of Helsinki. Written informed consent was obtained from all participants. Human tibial plateau specimens were collected from three patients with end-stage knee osteoarthritis (KOA) undergoing total knee arthroplasty at the Third Hospital of Hebei Medical University. For each patient, osteochondral blocks were harvested from both the medial and lateral tibial plateaus. Lateral plateau regions with preserved cartilage were designated as less-affected areas, whereas medial plateau regions with severe cartilage loss were designated as severely affected areas. Specimens were fixed in 4% paraformaldehyde, decalcified, and embedded in paraffin for subsequent immunohistochemical staining.

### Mice

*Piezo1^f/f^* mice (No. 029213; Jackson Laboratory), *OcnCreER^T2^* mice (No. 210445; Shanghai Model Organisms Center), *JAK2^f/f^* mice (No. 2102177; Shanghai Model Organisms Center), and *Rosa26^mTmG^* mice (No. C001192; Cyagen Biosciences) were used in this study. To obtain conditional knockout models, *Piezo1^f/f^* or *JAK2^f/f^* mice were bred with *OcnCreER^T2^* mice, yielding *OcnCreER^T2^; Piezo1^f/f^* and *OcnCreER^T2^; JAK2^f/f^*, which served as inducible conditional knockout (iCKO) models for Piezo1 and JAK2, respectively. These strains were further bred with *Rosa26^mTmG^* reporter mice to obtain *OcnCreER^T2^; Piezo1^f/f^; Rosa26^mTmG^* and *OcnCreER^T2^; JAK2^f/f^; Rosa26^mTmG^* lines. All animals were kept on a C57BL/6J background and reared under specific pathogen-free (SPF) facilities. To exclude potential effects of genetic background, CreERT2-negative littermates were used as controls in all experiments. Tamoxifen (TAM, T5648; Sigma-Aldrich) was prepared in pre-heated corn oil (37 °C) and given intraperitoneally at 75 mg/kg daily for one week prior to surgery, to induce osteoblast-specific activation of Cre recombinase. All groups, including both control and experimental mice, received the same TAM regimen. Genotyping of all strains was performed by PCR using specific primer sets as listed in Supplementary Table S1. To validate Cre recombinase activity in *Rosa26^mTmG^* mice, knee joint specimens were rapidly decalcified within 3 days, incubated overnight in 30% sucrose, and embedded in optimal cutting temperature (OCT) compound (SAKURA, 4583). Frozen samples were sectioned at 10 μm, re-warmed, and washed twice with TBS containing 0.1% Tween-20. Nuclei were counterstained with DAPI mounting medium (VECTOR, H-1200-10), and fluorescent images were acquired using an Olympus microscope (Tokyo, Japan).

### DMM surgery

Mice of various genotypes at 12 weeks of age were anesthetized with isoflurane. The skin around both knees was shaved and disinfected with povidone iodine solution. DMM surgery was performed as previously described 36. A skin incision was placed along the medial border of the patellar ligament. The fat pad overlying the anterior horn of the medial meniscus was gently separated using microsurgical forceps to expose the medial joint cavity. The ligament between the anterior horn of the medial meniscus and the tibial plateau was severed with a microscalpel. In sham-operated mice, the joint capsule was cut without disturbing the medial meniscus.

### AG490 injection

Wild-type (WT) DMM mice were assigned to two groups: one group received AG490 (MCE, HY-12000; 5 mg/kg, intraperitoneally) once daily for 6 weeks, and the control group was treated with vehicle under the same regimen.

### Calcein-Alizarin red S labeling

Ten days and three days before sacrifice, mice received intraperitoneal injections of calcein (Sigma, C0875-5G; 20 mg/kg) and alizarin red S (Sigma, A5533-25G; 40 mg/kg). Tibiae were harvested and fixed in 4% paraformaldehyde for 48 h. Following fixation, samples were processed by undecalcified dehydration and embedded in PMMA. Sections (10 µm) were prepared from PMMA blocks and imaged under fluorescence microscopy. Mineral apposition rate (MAR) in subchondral trabeculae beneath cartilage lesions was determined at one representative site per mouse in each group.

### Open field test (OFT)

Six weeks post-DMM, mice were acclimated (≥ 30 min) and tested in a 50 × 50 cm arena for 5 min to assess spontaneous locomotion. Total distance, mean speed, relative activity, and freezing time were automatically recorded and analyzed in EthoVision XT (Noldus).

### Rotarod assay

The accelerating rotarod assay was carried out 6 weeks after DMM; mice were pre-trained for 2 days. On the test day, each mouse was placed on an accelerating rotarod (Anhui Zhenghua Biological Instrument Equipment Co., Ltd., ZH-600B), with the rotation speed ramped from 4 revolutions per minute (RPM) to 40 RPM over 270 seconds. Latency to fall was recorded, capped at 300 s per trial.

### Micro-computed tomography (Micro-CT) analysis

Micro-CT was employed to assess tibial subchondral bone microarchitecture under different experimental conditions. Specimens were fixed (4% PFA, 48 h) and scanned (100 kV, 98 μA; voxel size 12 μm) on a Bruker Skyscan 1176 system. Reconstruction used NRecon v1.7 and segmentation DataViewer v1.5. The region of interest (ROI) was delineated on coronal slices with the subchondral bone plate (SBP) excluded, retaining only subchondral trabecular bone (STB) for analysis; medial and lateral STB were segmented separately. Mineralized tissue was segmented using a global grayscale threshold (CT range: 170-255) to binarize bone from non-bone. CTAn v1.9 was used to compute bone volume fraction (BV/TV), bone mineral density (BMD), trabecular thickness (Tb.Th), trabecular number (Tb.N), and trabecular separation (Tb.Sp), as well as geometric parameters of the tibial plateau, including plateau width, plateau thickness, and tibial plateau epiphyseal angle (TPEA) 37. Coronal images of tibial subchondral bone were generated using CTVox v3.3.

### Histological analysis

Decalcification of knee specimens was completed within 3 days with a rapid decalcification solution (PVB-3003, PhenoVision Bio). Specimen processing involved dehydration in graded ethanol and xylene, followed by paraffin embedding. Mouse knee joints were coronally sectioned at 4 μm thickness with a microtome (Leica RM2245, Germany). In total, 13-16 sequential sections were collected at 80 μm intervals to permit histological evaluation of the whole articular surface, identification of the most severe lesions, and determination of the overall extent of tissue damage. Sections underwent Safranin-O/Fast Green staining (SO&FG, Solarbio, G1371, China), and images of coronal knee joints were acquired using an Olympus optical microscope (Tokyo, Japan). Cartilage damage was evaluated in a blinded fashion according to the OARSI histopathology scoring system for mice (0-6 scale), with the maximum score across all sections representing KOA severity 38.

### Immunohistochemical (IHC) staining

Paraffin sections underwent heat-induced antigen retrieval in a 55 °C water bath overnight. Endogenous peroxidase activity was quenched with 3% H₂O₂ for 30 min. Blocking was performed using 10% goat serum for 1 h at room temperature, after which sections were exposed overnight at 4 °C to the following primary antibodies: anti-Piezo1 (Proteintech, 15939-1-AP, 1:100), anti-p-JAK2 (Abcam, ab32101, 1:100), anti-p-STAT3 (Abcam, ab76315, 1:100), anti-RUNX2 (Abcam, ab192256, 1:500), and anti-Osteocalcin (Takara, M188, 1:100). The following day, sections were treated for 1 h at room temperature with HRP-conjugated secondary antibodies: goat anti-rabbit IgG H&L (Abcam, ab205718, 1:10000) and goat anti-rat IgG H&L (Beyotime, A0192, 1:100). Staining was visualized using a DAB substrate kit (ZSGB-BIO, ZLI-9018). Nuclei were counterstained with Mayer's hematoxylin (Solarbio, G1080). Images were acquired microscopically, and quantitative histomorphological analyses were performed with ImageJ software.

### Immunofluorescence (IF) staining

To assess Piezo1 or JAK2 deletion efficiency in *OcnCreER^T2^* mice, frozen knee joint sections were processed as previously described. After equilibration at room temperature, sections underwent permeabilization in 0.25% Triton X-100/TBS for 10 min, followed by blocking with 10% goat serum at room temperature for 1 h. They were subsequently exposed overnight at 4 °C to primary antibodies, including anti-Piezo1 (Invitrogen, MA5-32876, 1:100), anti-p-JAK2 (Abcam, ab32101, 1:100), anti-OCN (Santa Cruz, sc-365797, 1:50), and anti-OCN (Affinity, DF12303, 1:100). After washing, the sections were incubated with secondary antibodies for 1 h at room temperature: Alexa Fluor 568 goat anti-mouse IgG2a (Invitrogen, A21134, 1:1000) and Alexa Fluor 488 goat anti-rabbit IgG H&L (Abcam, ab150077, 1:1000). Nuclear staining was performed with DAPI (Vector, H-1200-10), and fluorescence images were collected using an Olympus microscope (Tokyo, Japan).

### Cell culture

The MC3T3-E1 pre-osteoblastic cell line, derived from the calvaria of mouse embryos, was obtained from Cyagen Biosciences. Cells were maintained in α-minimum essential medium (α-MEM, Gibco, C11095500BT) containing 10% fetal bovine serum (FBS, Gibco, A5256701) and 1% penicillin-streptomycin (Gibco, 15140122) under standard conditions (37 °C, 5% CO₂, humidified atmosphere). Culture medium was refreshed every 2-3 days, and cells were subcultured at 80-90% confluence using 0.25% trypsin-EDTA (Gibco, 25200-056) before reseeding at a ratio of 1:3. For genome editing, a single-guide RNA (sgRNA; target sequence: 5′-AGCCCATAACAGCAGGATGCA-3′; PAM: GGG) targeting the Piezo1 locus was designed and cloned for CRISPR/Cas9-mediated knockout. Cas9/sgRNA plasmids were introduced into MC3T3-E1 cells by electroporation. Single-cell-derived colonies were subsequently expanded, and Piezo1 deletion was screened by PCR and further validated by Sanger sequencing (Fig. S1). The MC3T3-E1 clone used in this study harbored biallelic indels at the sgRNA cut site (Δ35 bp and Δ62 bp), as shown in Fig. S1. Because both alleles are present in the same clone, Sanger chromatograms around the cut site can show mixed/out-of-phase peaks downstream of the indel. The primer sequences used for PCR and sequencing are provided in Table S2.

### Osteogenic differentiation and staining assays

For osteogenic induction, MC3T3-E1 and Piezo1-KO MC3T3-E1 cells were cultured in a commercial osteogenic differentiation medium (Cyagen, MUXMT-90021). After 7 days of induction, cells were washed with PBS and fixed in 4% paraformaldehyde. Alkaline phosphatase (ALP) staining was performed using an ALP staining kit (Beyotime, C3206) according to the manufacturer's instructions, and ALP staining intensity was quantified using ImageJ software. After 14 days of induction, mineralized matrix deposition was assessed by Alizarin Red S (ARS) staining using a commercial kit (Solarbio, G3283). Following staining, bound ARS dye was extracted and the optical density at 560 nm was measured to obtain a semi-quantitative estimate of mineralization.

### Cyclic tensile strain (CTS) treatment

After 5 days of osteogenic induction in collagen I-coated BioFlex 6-well culture plates (Flexcell International Corp, Burlington, NC, USA), cells were transferred to a Flexcell FX-5000 Tension System (Flexcell International) and subjected to cyclic sinusoidal tensile strain (10%, 0.5 Hz). Cells were collected for analysis after 8 hours of treatment.

### Drug treatments in control and Piezo1-KO MC3T3-E1 cells

For pharmacological assays, control and Piezo1-KO MC3T3-E1 cells were seeded at 2.0 × 10^5^ cells per well and treated under the following conditions: JAK2 inhibition with 10 µM AG490 (MCE, HY-12000) or STAT3 inhibition with 5 µM Stattic (Santa Cruz, sc-202818) for 24 h in BioFlex plates, and JAK2 activation with 20 µM Broussonin E (MCE, HY-N2963) for 3 h in 6-well plates. Following drug exposure, cells were harvested for subsequent analyses.

### RT-PCR analysis

Total RNA was isolated from MC3T3-E1 cells using TRIzol reagent (Invitrogen, 15596026). Complementary DNA (cDNA) was synthesized with the GoScript Reverse Transcription Mix and Oligo dT primers (Promega, Madison, WI, USA). Quantitative PCR was carried out with PowerTrack SYBR Green Master Mix on an Applied Biosystems 7500 Real-Time PCR System (Thermo Scientific, UT, USA). For data processing, expression levels were normalized to the control group, and relative mRNA abundance for each sample was determined using the 2^-ΔΔCT^ method. All primers were obtained from Sangon Biotech, and their sequences are listed as follows:

*Runx2*: Forward: GAGTCAGATTACAGATCCCA; Reverse: TGGCTCTTCTTACTGAGAGA. *Alpl*: Forward: ATCTTTGGTCTGGCTCCCATG; Reverse: TTTCCCGTTCACCGTCCAC. *Gapdh*: Forward: ATGTGTCCGTCGTGGATCTGA; Reverse: ATGCCTGCTTCACCACCTTCTT.

### Western blotting

Total protein was isolated from MC3T3-E1 cells using a lysis buffer containing protease and phosphatase inhibitor cocktails (Beyotime, P1045, P0013B). Protein concentrations were quantified by the BCA method with a commercial kit (ThermoFisher, 23225). Equal protein amounts (30 μg) were resolved on 4-20% SDS-PAGE gels (Geneticpit, M00657) and subsequently blotted onto polyvinylidene fluoride (PVDF) membranes (EMD Millipore, USA). Membranes were blocked with Quick Blocking Buffer (Solarbio, SW3012) for 15 min at room temperature and then incubated with primary antibodies overnight at 4 °C. Antibodies included RUNX2 (Abcam, ab236639; 1:5000), OCN (Affinity, DF12303; 1:1000), Piezo1 (Proteintech, 15939-1-AP; 1:1000), JAK2 (ThermoFisher, MA5-44619; 1:1000), p-JAK2 (Abcam, ab32101; 1:1000), STAT3 (Proteintech, 60199-1-Ig; 1:1000), p-STAT3 (Abcam, ab76315; 1:1000), and GAPDH (Abcam, ab181602; 1:15000). Following washes, the membranes were exposed to HRP-labeled secondary antibodies for 1 h at ambient temperature. Signal detection was performed with a chemiluminescence substrate kit (ZETA, 310208), and images were captured with a Bio-Rad imaging platform (California, USA). Band intensities were quantified using ImageJ software.

### Nuclear and cytoplasmic protein extraction

For nuclear-cytoplasmic fractionation, MC3T3-E1 cells were subjected to 10% cyclic tensile strain (CTS, 0.5 Hz) for 0, 4, or 8 h. At each time point, cells were harvested, and nuclear and cytoplasmic protein fractions were isolated using a Nuclear and Cytoplasmic Protein Extraction Kit (Solarbio, EX1470) according to the manufacturer's instructions. Protein concentrations were determined using a BCA assay, and equal amounts of nuclear or cytoplasmic proteins were subjected to Western blot analysis. The distribution of total STAT3 between nuclear and cytoplasmic fractions was evaluated using an anti-STAT3 antibody (Proteintech, 60199-1-Ig; 1:1000), with Histone H3 (Proteintech, 17168-1-AP; 1:1000) and GAPDH (Abcam, ab181602; 1:15000) serving as nuclear and cytoplasmic loading controls, respectively.

### Cell immunofluorescence staining

MC3T3-E1 cells were fixed in 4% paraformaldehyde for 15 min and then permeabilized with 0.3% Triton X-100 (Solarbio, T8200) for an additional 15 min. After blocking with 10% bovine serum albumin (BSA) for 1 h, the samples were incubated overnight at 4 °C with primary antibodies: RUNX2 (Abcam, ab236639; 1:500), p-JAK2 (Abcam, ab32101; 1:100), and p-STAT3 (Abcam, ab76315; 1:100). After washing, sections were exposed for 1 h at room temperature to secondary antibodies (Abcam, ab150062; 1:1000). Nuclei were counterstained with DAPI (VECTOR, H-1200-10). Fluorescence images were obtained using an Olympus BX53 microscope (Japan).

### Intracellular calcium measurement

Cells were digested with 0.25% trypsin-EDTA (ZETA, BM0002-100) and centrifuged at 1500 rpm for 5 min. After washing with PBS, the pellet was resuspended in 2.5 μM Fluo-4 AM (Beyotime, S1060) and incubated at 37 °C for 20 min. Cells were then stimulated with 10 μM Yoda1 (MCE, HY-18723), 20 μM BAPTA-AM (MCE, HY-100545), or 5 μM ionomycin (MCE, HY-13434). Fluorescence signals were measured on a flow cytometer (BD FACSCelesta, NJ, USA) with excitation at 488 nm. Data were plotted against area (FITC-A) and analyzed using FlowJo software (Version 10.9.0, FlowJo LLC, Ashland, OR, USA).

### Statistical analysis

All data are expressed as mean ± standard deviation (SD). Each *in vitro* experiment was independently repeated at least three times, and representative outcomes are presented. Comparisons between two groups were carried out using Student's *t*-test, whereas one-way or two-way ANOVA was employed for multiple-group analyses. A threshold of *p* < 0.05 was taken to indicate statistical significance. Statistical calculations were conducted with GraphPad Prism version 9.5 (GraphPad Software, San Diego, CA, USA).

## Results

### Piezo1 is upregulated in medial tibial plateau subchondral bone during KOA progression

To clarify the potential involvement of Piezo1 in KOA, we first assessed its expression in human tibial plateau specimens. Immunohistochemistry revealed stronger Piezo1 staining in the medial subchondral bone than in the paired lateral region in all three matched cases (Fig. S2).

**Figure 1:**
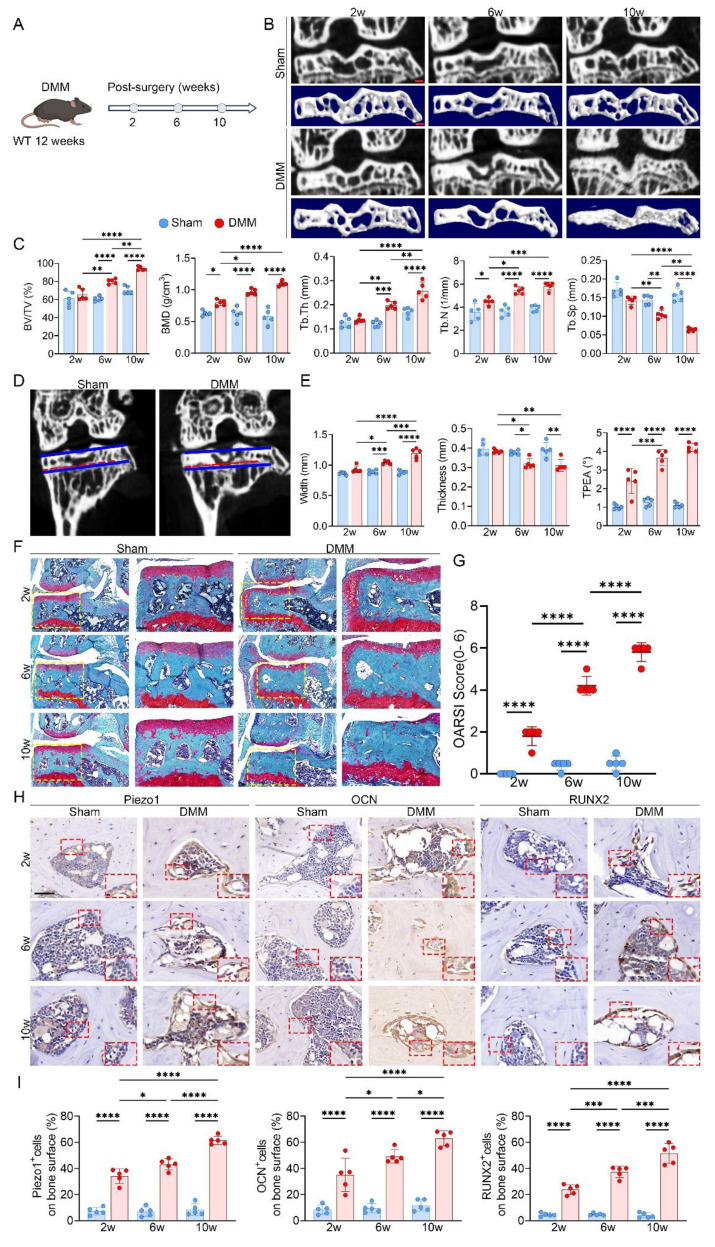
Progressive uneven tibial plateau settlement with concomitant subchondral bone sclerosis, cartilage degeneration, and Piezo1 upregulation in DMM-induced KOA. (A) Experimental scheme illustrating the timeline following DMM surgery in 12-week-old WT mice; (B) Representative high resolution coronal micro-CT images (top) and corresponding 3D reconstructions (bottom) of knee joints from sham and DMM mice (scale bar = 500 μm); (C) Quantitative analysis of medial subchondral bone parameters, including BV/TV, BMD, Tb.Th, Tb.N, and Tb.Sp, in sham and DMM groups (n = 5 mice per group); (D) Coronal micro-CT images showing measurement of TPEA, defined as the angle between the tibial plateau and the proximal epiphyseal line, in sham and DMM mice; (E) Quantification of medial tibial plateau width, medial tibial plateau thickness, and TPEA in tibial subchondral bone (n = 5 mice per group); (F) Representative SO&FG staining of knee joints from sham and DMM mice (scale bar = 200 μm); (G) OARSI scores evaluating cartilage damage in sham and DMM groups (n = 5 mice per group); (H) Representative immunohistochemical staining of Piezo1, OCN, and RUNX2 in subchondral bone; red boxes indicate areas shown at higher magnification, and red arrows highlight representative positive cells (scale bar = 50 μm; enlarged views, 5 μm); (I) Quantification of OCN⁺, Piezo1⁺, and RUNX2⁺ cell percentages on the medial tibial subchondral bone surface (n = 5 mice per group). *p < 0.05; **p < 0.01; ***p < 0.001; **** p < 0.0001.

To model KOA *in vivo*, we established a DMM mouse model and performed longitudinal micro-CT scanning at 2, 6, and 10 weeks after surgery (Fig. 1A, B). At 2 weeks, there were no detectable differences in medial subchondral bone architecture between the DMM and sham groups. By 6 weeks, however, DMM mice exhibited evident remodeling in the medial compartment, with increases in BV/TV, BMD, Tb.Th and Tb.N, along with a marked reduction in Tb.Sp (Fig. 1C). During this stage, the medial tibial plateau widened and thinned, accompanied by an increased TPEA due to medial deviation of the mechanical axis (Fig. 1D, E). These alterations were further aggravated by 10 weeks, with pronounced trabecular disorganization reflecting progressive subchondral sclerosis.

Structural analyses revealed that medial subchondral bone underwent more severe changes than the lateral side, showing significantly higher ΔBV/TV, ΔBMD, ΔTb.Th, and ΔTb.N, as well as more obvious decreases in ΔTb.Sp, indicating accelerated sclerosis in the medial region (Fig. S3A). Morphologically, the medial tibial plateau displayed a greater loss in thickness relative to the lateral plateau, supporting that uneven overload promotes regional tibial collapse (Fig. S3B). SO&FG staining showed progressive medial cartilage erosion, depletion of proteoglycans, and higher OARSI scores, together with increased trabecular density beneath the cartilage (Fig. 1F, G). Immunohistochemistry further demonstrated stepwise upregulation of RUNX2, OCN, and Piezo1 in the medial subchondral bone (Fig. 1H, I). Since cartilage destruction was most evident at 10 weeks, representing advanced KOA, the 6-week time point was selected for subsequent mechanistic studies as it better reflected the active phase of subchondral bone remodeling.

### Osteoblast-specific Piezo1 deletion attenuates subchondral sclerosis and cartilage damage

**Figure 2:**
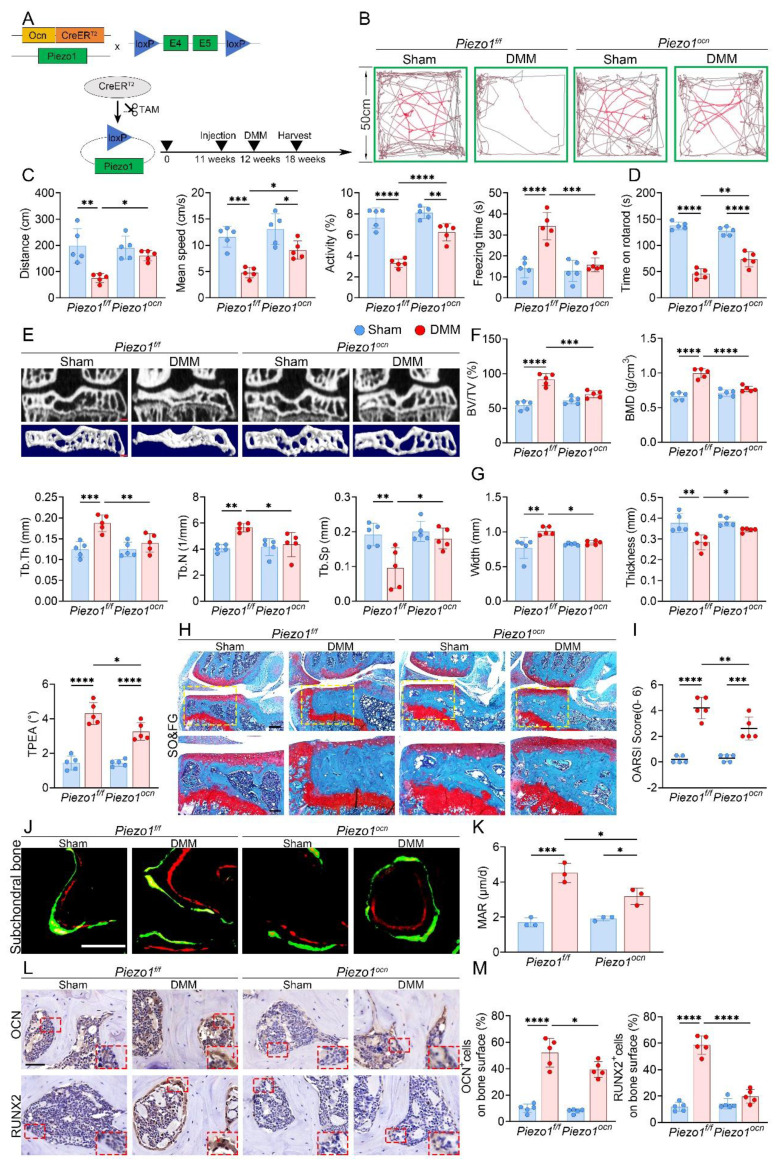
Osteoblast-specific Piezo1 deletion attenuates subchondral bone sclerosis and KOA progression. (A) Schematic illustrating the generation of osteoblast-specific Piezo1 conditional knockout mice using a TAM-inducible *CreER^T2^* system driven by the osteocalcin promoter in 12 week old *Piezo1^f/f^* and *Piezo1^ocn^* mice; (B) Representative locomotor traces from the OFT showing movement patterns in sham and DMM groups of P*iezo1^f/f^* and *Piezo1^ocn^* mice; (C) Quantitative analysis of behavioral parameters including total distance traveled, mean speed, relative activity, and freezing time (n = 5 mice per group); (D) Rotarod performance test showing the time each mouse remained on the rotating rod (n = 5 mice per group); (E) Representative micro-CT images showing coronal sections of the tibial subchondral bone in the upper row and corresponding 3D reconstructions in the lower row from sham and DMM mice of both genotypes (scale bar = 500 μm); (F) Quantitative assessment of subchondral bone microarchitecture, including BV/TV, BMD, Tb.Th, Tb.N, and Tb.Sp (n = 5 mice per group); (G) Quantification of medial tibial plateau width, medial tibial plateau thickness and TPEA in tibial subchondral bone (n = 5 mice per group); (H) Representative SO&FG staining of knee joint sections from *Piezo1^f/f^* and *Piezo1^ocn^* mice following sham or DMM surgery (scale bar = 200 μm); (I) Quantification of cartilage degeneration using OARSI scoring (n = 5 mice per group); (J) Representative fluorescent images of calcein and alizarin red S double labeling in the tibial subchondral bone of sham and DMM mice in both genotypes (scale bar = 50 μm); (K) Quantification of MAR based on fluorochrome labeling (n = 5 mice per group); (L) Representative immunohistochemical staining of OCN and RUNX2 in the tibial subchondral bone; red boxes indicate areas shown at higher magnification, and red arrows highlight representative positive cells (scale bar = 50 μm; enlarged views, 5 μm); (M) Quantification of OCN⁺ and RUNX2⁺ cell percentages on the medial tibial subchondral bone surface (n = 5 mice per group). *p < 0.05; **p < 0.01; ***p < 0.001; ****p < 0.0001.

To explore the role of Piezo1 in osteoblasts, we generated *OcnCreER^T2^*; *Piezo1^f/f^; Rosa26^mTmG^* reporter mice and verified Cre recombination in subchondral osteoblasts by the color switch from mTomato to mGFP following TAM administration (Fig. S4A, B). Subsequently, *OcnCreER^T2^; Piezo1^f/f^* mice were used, in which Piezo1 deletion was induced after one week of TAM injections and confirmed by double immunofluorescence staining for OCN and Piezo1 in subchondral bone (Fig. S4C, D). The experimental design and knockout strategy are illustrated in Fig. 2A. At 6 weeks after DMM, both OFT and rotarod assays demonstrated enhanced locomotor activity and motor performance in Piezo1 iCKO mice relative to controls (Fig. 2B-D). Micro-CT analyses further showed that subchondral sclerosis was alleviated in iCKO mice, evidenced by reduced BV/TV, BMD, Tb.Th and Tb.N, together with increased Tb.Sp (Fig. 2E, F). Morphologically, the medial tibial plateau exhibited improved structural features, including thicker trabeculae, less widening, and reduced TPEA, indicating partial recovery from settlement of the medial tibial plateau (Fig. 2G). Moreover, the disparity between medial and lateral compartments was less evident in iCKO mice, suggesting attenuation of uneven tibial plateau settlement (Fig. S4E, F). SO&FG revealed reduced cartilage degeneration and lower OARSI scores (Fig. 2H, I). Calcein-Alizarin Red labeling showed decreased MAR in subchondral trabeculae (Fig. 2J, K). In addition, fewer RUNX2- and OCN-positive osteoblasts were detected in the medial subchondral bone, consistent with reduced osteogenic activity (Fig. 2L, M).

### Piezo1 dependent mechanical stimulation enhances osteoblast differentiation

**Figure 3:**
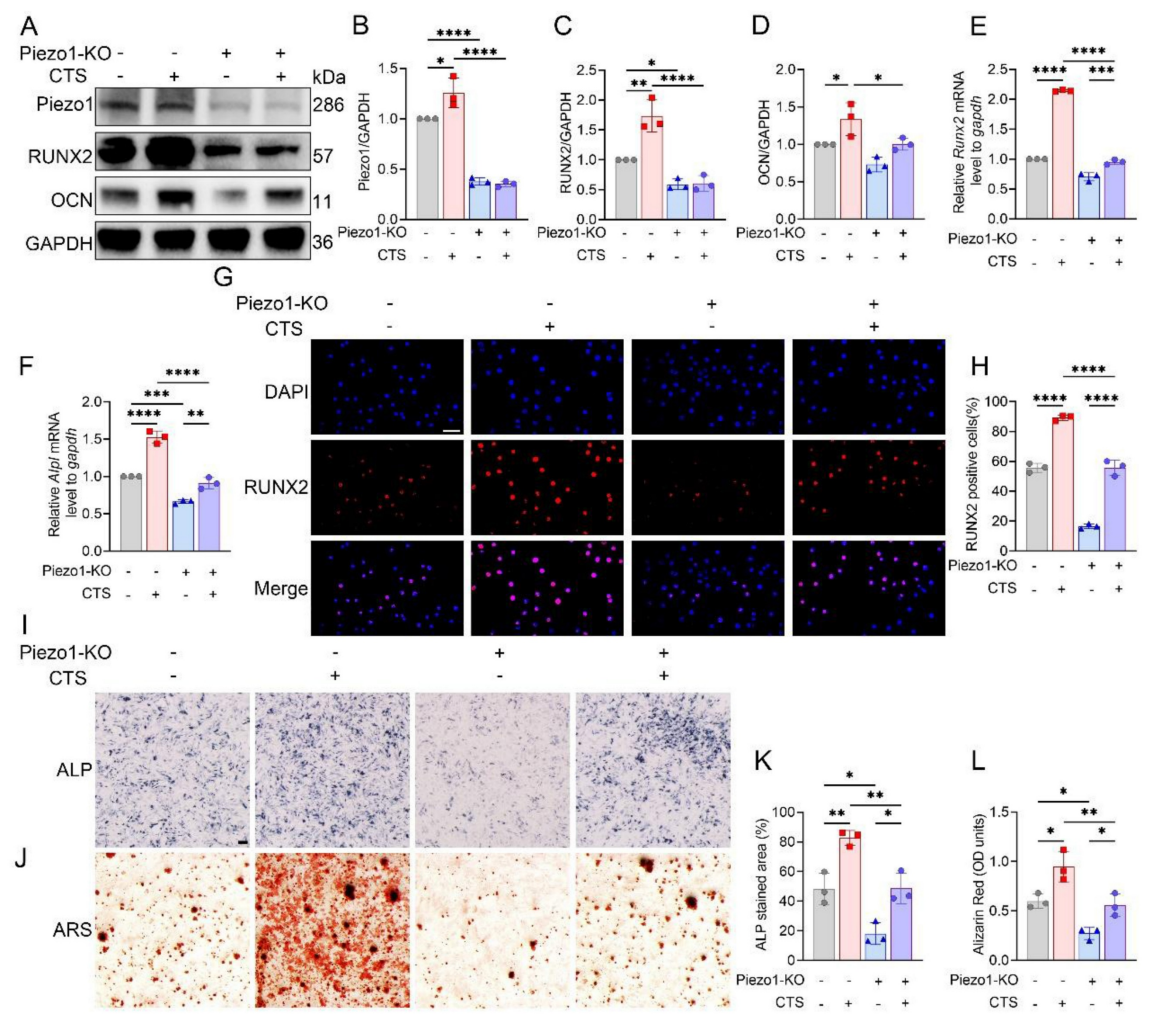
Piezo1 mediates mechanical force induced osteogenic differentiation *in vitro*. (A) Western blot analysis of Piezo1, RUNX2, and OCN protein expression in MC3T3-E1 and Piezo1-KO MC3T3-E1 cells with or without CTS treatment (n = 3 per group). GAPDH was used as the loading control. (B) Quantification of Piezo1 protein levels normalized to GAPDH (n = 3 per group). (C) Quantification of RUNX2 protein levels normalized to GAPDH (n = 3 per group). (D) Quantification of OCN protein levels normalized to GAPDH (n = 3 per group). (E) Relative mRNA expression of *Runx2* detected by qPCR under each condition (n = 3 per group). (F) Relative mRNA expression of *Alpl* detected by qPCR under each condition (n = 3 per group). (G) Representative immunofluorescence staining of RUNX2 and DAPI in MC3T3-E1 and Piezo1-KO MC3T3-E1 cells under different treatments (scale bar = 50 μm). (H) Quantification of RUNX2-positive cells based on immunofluorescence staining (n = 3 per group). (I) Representative ALP staining to assess early osteogenic differentiation in control and Piezo1-KO MC3T3-E1 cells with or without CTS (scale bar = 500 μm). (J) Representative ARS staining to evaluate matrix mineralization in control and Piezo1-KO MC3T3-E1 cells with or without CTS (scale bar = 500 μm). (K) Quantification of ALP-stained area (%) (n = 3 per group). (L) Quantification of Alizarin Red (OD units) (n = 3 per group). * *p* < 0.05; ** *p* < 0.01; *** *p* < 0.001; **** p < 0.0001.

Efficient knockout of Piezo1 was confirmed in MC3T3-E1 Piezo1-KO cells (Fig. 3A, B). Following CTS stimulation, control cells showed increases in *Runx2* and *Alpl* mRNA levels, along with increased RUNX2 and OCN protein expression, whereas these responses were blunted in Piezo1-KO cells (Fig. 3C-F). Immunofluorescence further demonstrated robust nuclear accumulation of RUNX2 in CTS-treated control cells, which was markedly suppressed in Piezo1-KO cells (Fig. 3G, H). In parallel, ALP and ARS staining confirmed that CTS promoted osteogenic differentiation and matrix mineralization in control cells, but these effects were diminished under Piezo1 deficiency (Fig. 3I-L). Together, these findings indicate that Piezo1 is indispensable for CTS-induced osteoblast differentiation.

### Osteoblast-specific JAK2 deletion reduces subchondral bone sclerosis and cartilage damage in KOA

**Figure 4:**
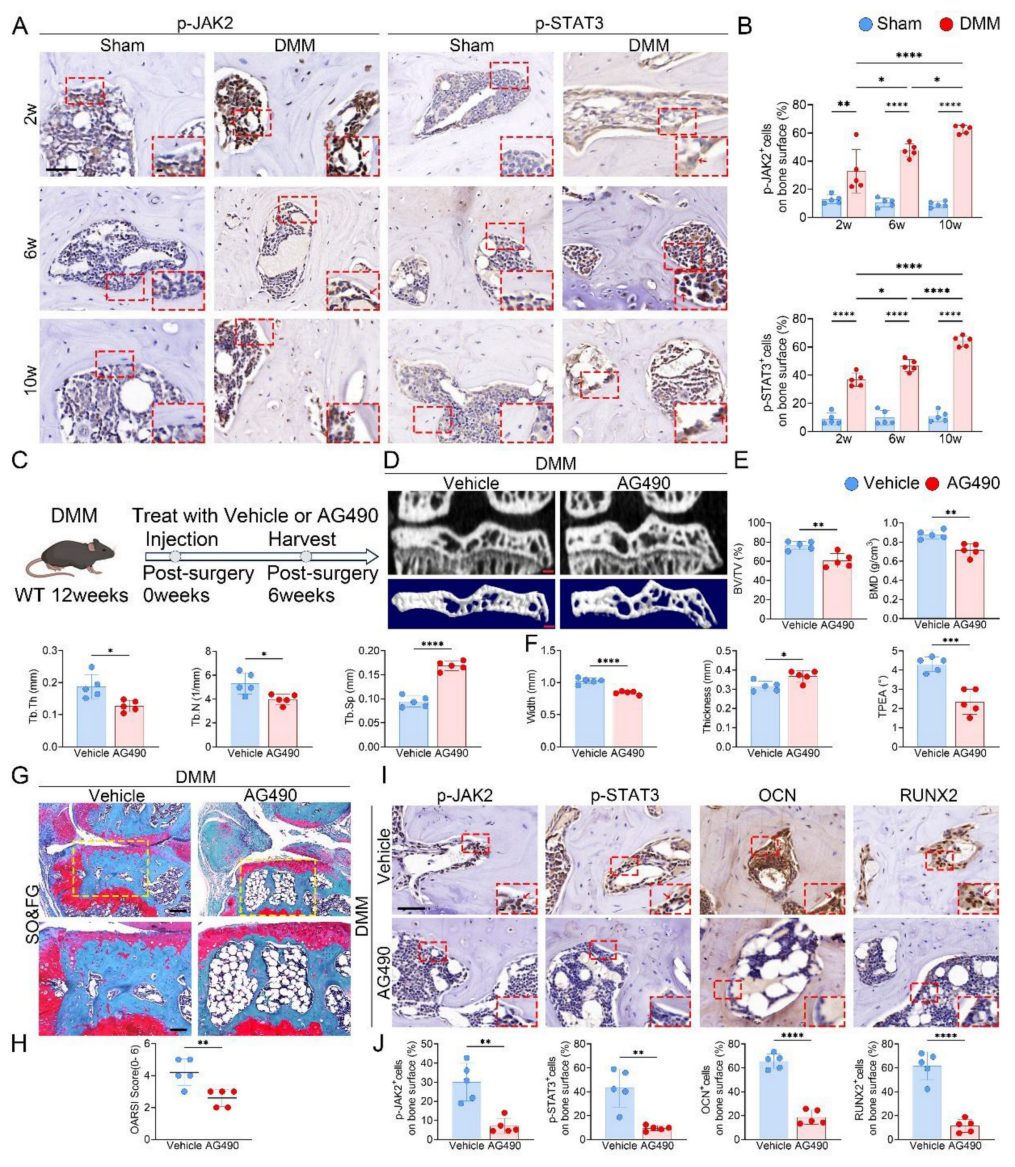
Inhibition of JAK2/STAT3 signaling attenuates subchondral bone sclerosis and osteogenic marker expression during KOA progression. (A) Representative immunohistochemical staining of p-JAK2 and p-STAT3 in the medial tibial subchondral bone of sham and DMM mice at 2, 6 and 10 weeks post-surgery; red boxes indicate areas shown at higher magnification, and red arrows highlight representative positive cells (scale bar = 50 μm; enlarged views, 5 μm); (B) Quantification of the percentages of p-JAK2⁺ and p-STAT3⁺ cells in the medial tibial subchondral bone (n = 5 mice per group); (C) Schematic of the AG490 treatment regimen. DMM was performed at 12 weeks of age, followed by intraperitoneal AG490 or vehicle for 6 weeks; mice were harvested 6 weeks post-surgery; (D) Representative high resolution coronal micro-CT images (top) and corresponding 3D reconstructions (bottom) of tibial subchondral bone in DMM mice treated with vehicle or AG490 (scale bar = 500 μm); (E) Quantitative analysis of subchondral bone microarchitecture, including BV/TV, BMD, Tb.Th, Tb.N, and Tb.Sp (n = 5 mice per group); (F) Quantification of medial tibial plateau width, medial tibial plateau thickness and TPEA (n = 5 mice per group); (G) Representative SO&FG staining of knee joint sections from DMM mice treated with vehicle or AG490 (scale bar = 200 μm); (H) OARSI histological scores quantifying cartilage degeneration in each group (n = 5 mice per group); (I) Representative immunohistochemical staining of p-JAK2, p-STAT3, OCN and RUNX2 in the medial tibial subchondral bone from DMM mice treated with vehicle or AG490; red boxes indicate areas shown at higher magnification, and red arrows highlight representative positive cells (scale bar = 50 μm; enlarged views, 5 μm); (J) Quantification of p-JAK2⁺, p-STAT3⁺, OCN⁺ and RUNX2⁺ cell percentages on the medial tibial subchondral bone surface (n = 5 mice per group). *p < 0.05; **p < 0.01; ***p < 0.001; **** p < 0.0001.

In light of the increased Piezo1 expression in KOA subchondral bone, we next assessed the changes in JAK2-STAT3 signaling. In tibial plateau specimens from KOA patients, immunostaining for p-JAK2 and p-STAT3 was stronger in the medial subchondral bone than in the lateral side (Fig. S2). In DMM mice, time-course IHC staining further revealed progressive upregulation of p-JAK2 and p-STAT3 in the medial subchondral bone following surgery (Fig. 4A, B). To further evaluate the contribution of this pathway, DMM mice were administered the JAK2 inhibitor AG490 (Fig. 4C). Micro-CT analyses showed that AG490 markedly reduced BV/TV, BMD, Tb.Th and Tb.N, while elevating Tb.Sp and improving tibial plateau morphology (Fig. 4D-F). Histological staining confirmed that AG490 treatment attenuated cartilage degeneration, accompanied by reduced OARSI scores (Fig. 4G, H). IHC analysis further demonstrated decreased expression of p-JAK2, p-STAT3, OCN, and RUNX2 in the subchondral bone of AG490-treated mice (Fig. 4I, J).

**Figure 5:**
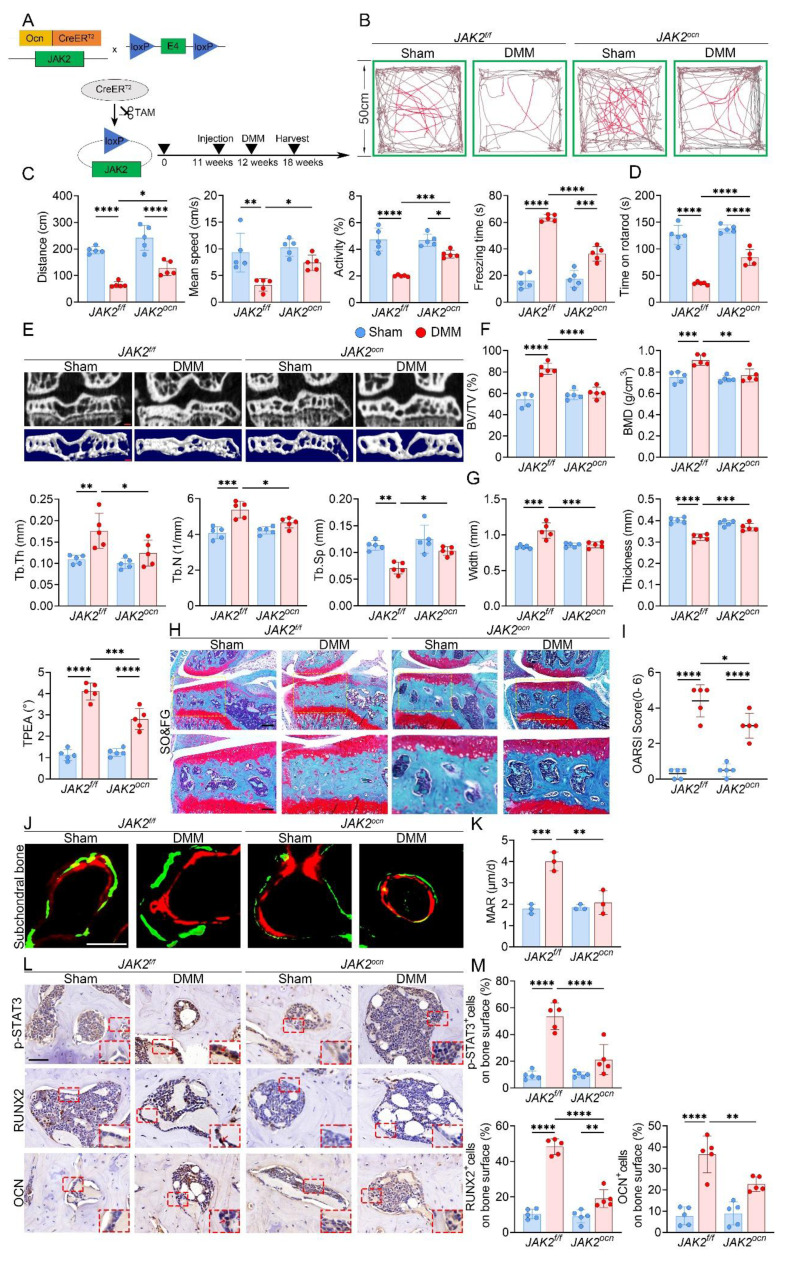
Conditional deletion of JAK2 in osteoblasts alleviates subchondral bone sclerosis and osteogenic marker expression during KOA progression. (A) Schematic illustrating the TAM induced JAK2 conditional knockout strategy in 12 week old *JAK2^f/f^* and* JAK2^ocn^* mice; (B) Representative locomotor traces from the OFT in sham and DMM groups of *JAK2^f/f^* and *JAK2^ocn^* mice; (C) Quantitative analysis of behavioral parameters including total distance traveled, mean speed, relative activity, and freezing time (n = 5 mice per group); (D) Rotarod performance test showing the time each mouse remained on the rotating rod (n = 5 mice per group); (E) Representative high resolution coronal micro-CT images (top) and corresponding 3D reconstructions (bottom) of tibial subchondral bone in sham and DMM mice with *JAK2^f/f^* or *JAK2^ocn^* genotype (scale bar = 500 μm); (F) Quantification of subchondral bone microarchitectural parameters, including BV/TV, BMD, Tb.Th, Tb.N, and Tb.Sp (n = 5 mice per group); (G) Quantification of medial tibial plateau width, medial tibial plateau thickness, and TPEA (n = 5 mice per group); (H) Representative SO&FG staining of knee joints from sham and DMM groups in* JAK2^f/f^* and *JAK2^ocn^* mice (scale bar = 200 μm); (I) OARSI histological scores quantifying cartilage degeneration (n = 5 mice per group); (J) Representative fluorescent images of calcein and alizarin red S double labeling in the tibial subchondral bone from sham and DMM mice in both genotypes (scale bar = 50 μm); (K) Quantification of mineral apposition rate based on double labeling analysis (n = 5 mice per group); (L) Representative immunohistochemical staining of p-STAT3, RUNX2, and OCN in the medial tibial subchondral bone from each group; red boxes indicate areas shown at higher magnification, and red arrows highlight representative positive cells (scale bar = 50 μm; enlarged views, 5 μm); (M) Quantification of p-STAT3⁺, OCN⁺ and RUNX2⁺ cell percentages on the medial tibial subchondral bone surface (n = 5 mice per group). *p < 0.05; **p < 0.01; ***p < 0.001; **** p < 0.0001.

The efficiency of osteoblast-specific JAK2 deletion was first validated by immunofluorescence staining for OCN and JAK2 (Fig. S5A). The knockout strategy and workflow are illustrated in Fig. 5A. At 6 weeks after DMM, both OFT and rotarod tests revealed enhanced locomotion and motor function in *JAK2^ocn^* mice compared with controls (Fig. 5B-D). Micro-CT analysis further indicated that JAK2 deletion markedly alleviated medial subchondral sclerosis, reflected by lower BV/TV, BMD, Tb.Th and Tb.N, along with increased Tb.Sp and improved plateau morphology (Fig. 5E-G). Consistently, disparities between medial and lateral compartments in trabecular parameters were also reduced in *JAK2^ocn^* mice (Fig. S5B, C). Histological analysis with SO&FG staining revealed reduced cartilage destruction and decreased OARSI scores (Fig. 5H, I). Calcein-Alizarin Red labeling further confirmed decreased MAR in the subchondral trabeculae of *JAK2^ocn^* mice (Fig. 5J, K). Moreover, fewer osteoblasts positive for RUNX2 and OCN were detected in the medial compartment, suggesting reduced osteogenic activity (Fig. 5L, M).

### JAK2/STAT3 signaling mediates mechanical stimulation-induced osteogenesis, and pharmacological activation restores osteogenesis in Piezo1-KO cells

**Figure 6:**
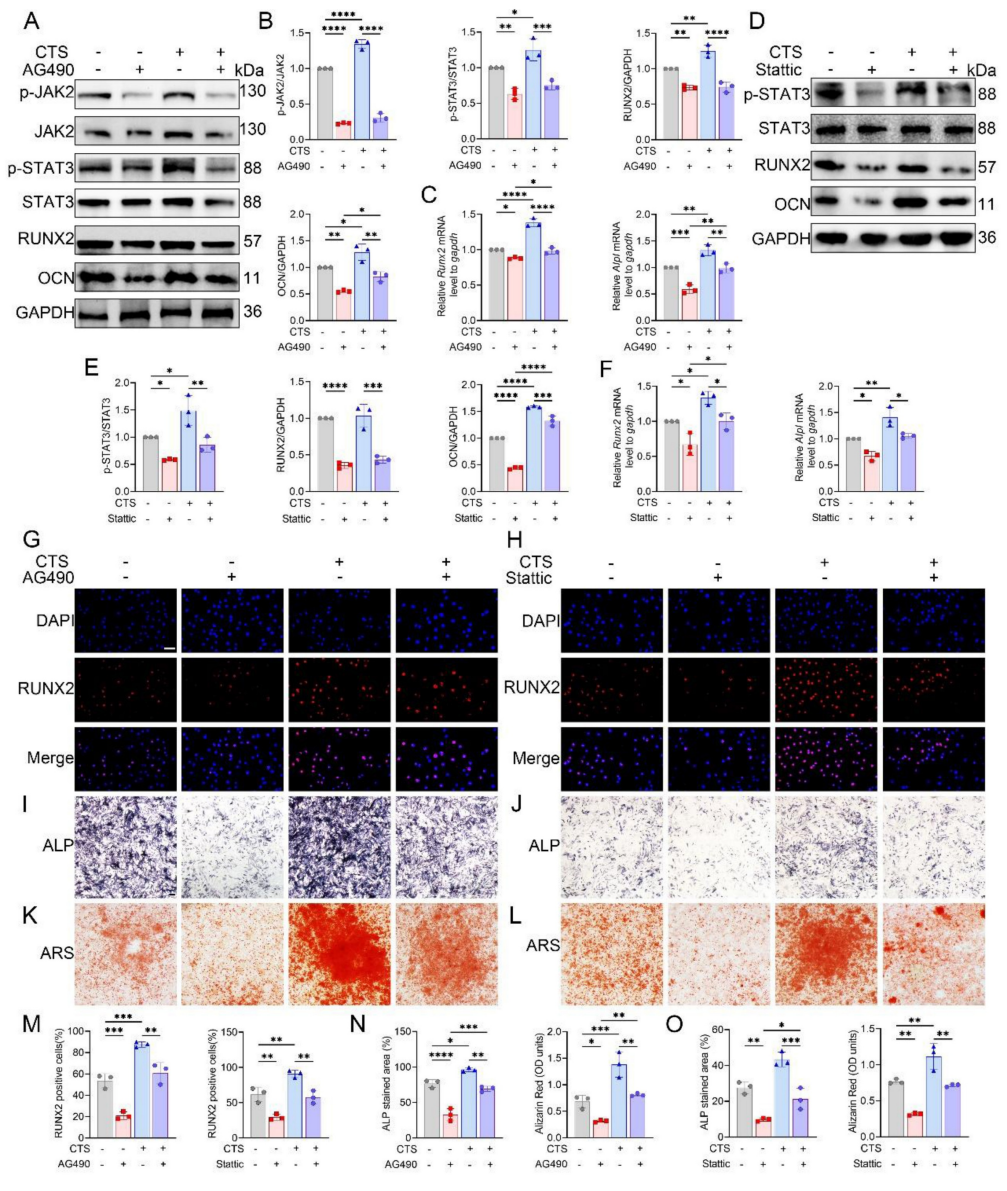
JAK2/STAT3 signaling mediates mechanical force induced osteogenic differentiation *in vitro*. (A) Western blot analysis of p-JAK2, total JAK2, p-STAT3, total STAT3, RUNX2, and OCN in MC3T3-E1 cells with or without CTS, in the presence or absence of AG490 (n = 3 per group). GAPDH was used as the loading control. (B) Quantification of p-JAK2 relative to total JAK2, p-STAT3 relative to total STAT3, and RUNX2 and OCN relative to GAPDH under AG490 conditions (n = 3 per group). (C) Relative mRNA expression of *Runx2* and *Alpl* assessed by qPCR under AG490 conditions (n = 3 per group). (D) Western blot analysis of p-STAT3, total STAT3, RUNX2, and OCN in MC3T3-E1 cells with or without CTS in the presence or absence of Stattic (n = 3 per group). (E) Quantification of p-STAT3 relative to total STAT3, and RUNX2 and OCN relative to GAPDH under Stattic conditions (n = 3 per group). (F) Relative mRNA expression of *Runx2* and *Alpl* assessed by qPCR following Stattic treatment (n = 3 per group). (G) Representative immunofluorescence staining of RUNX2 and DAPI with or without CTS under AG490 conditions (scale bar = 50 μm). (H) Representative immunofluorescence staining of RUNX2 and DAPI with or without CTS under Stattic conditions (scale bar = 50 μm). (I) Representative ALP staining with or without CTS under AG490 conditions (scale bar = 500 μm). (J) Representative ALP staining with or without CTS under Stattic conditions (scale bar = 500 μm). (K) Representative ARS staining with or without CTS under AG490 conditions (scale bar = 500 μm). (L) Representative ARS staining with or without CTS under Stattic conditions (scale bar = 500 μm). (M) Quantification of RUNX2-positive cells based on immunofluorescence under AG490 and Stattic conditions (n = 3 per group). (N) Quantification under AG490 conditions of ALP-stained area (%) and Alizarin Red (OD units) with or without CTS (n = 3 per group). (O) Quantification under Stattic conditions of ALP-stained area (%) and Alizarin Red (OD units) with or without CTS (n = 3 per group). *p < 0.05; **p < 0.01; ***p < 0.001; **** p < 0.0001.

CTS stimulation of MC3T3-E1 cells markedly promoted osteogenic differentiation. Pharmacological inhibition of JAK2 with AG490 diminished these effects, as reflected by reduced RUNX2 and OCN expression at both transcript and protein levels, together with decreased phosphorylation of JAK2 and STAT3 (Fig. 6A-C). Similarly, treatment with the STAT3 inhibitor Stattic suppressed osteogenesis, evidenced by lower RUNX2 and OCN levels and attenuated STAT3 phosphorylation (Fig. 6D-F). Consistent with these findings, immunofluorescence analysis revealed robust nuclear localization of RUNX2 after CTS, which was markedly reduced by AG490 or Stattic (Fig. 6G, H). Quantification further confirmed fewer RUNX2-positive cells under inhibitor treatment (Fig. 6M). Moreover, ALP and ARS staining demonstrated that CTS-induced increases in alkaline phosphatase activity and matrix mineralization were strongly blunted by AG490 and Stattic (Fig. 6I-L), with quantification supporting these observations (Fig. 6N, O). Collectively, these results suggest that both JAK2 and STAT3 are required for osteoblast responses to mechanical loading.

We further assessed the subcellular localization of STAT3 in response to mechanical stimulation. Nuclear-cytoplasmic fractionation followed by Western blot analysis showed that nuclear STAT3 protein levels were significantly increased after 4 and 8 h of CTS compared with 0 h (Fig. S6), indicating enhanced nuclear accumulation of STAT3 under mechanical loading.

**Figure 7:**
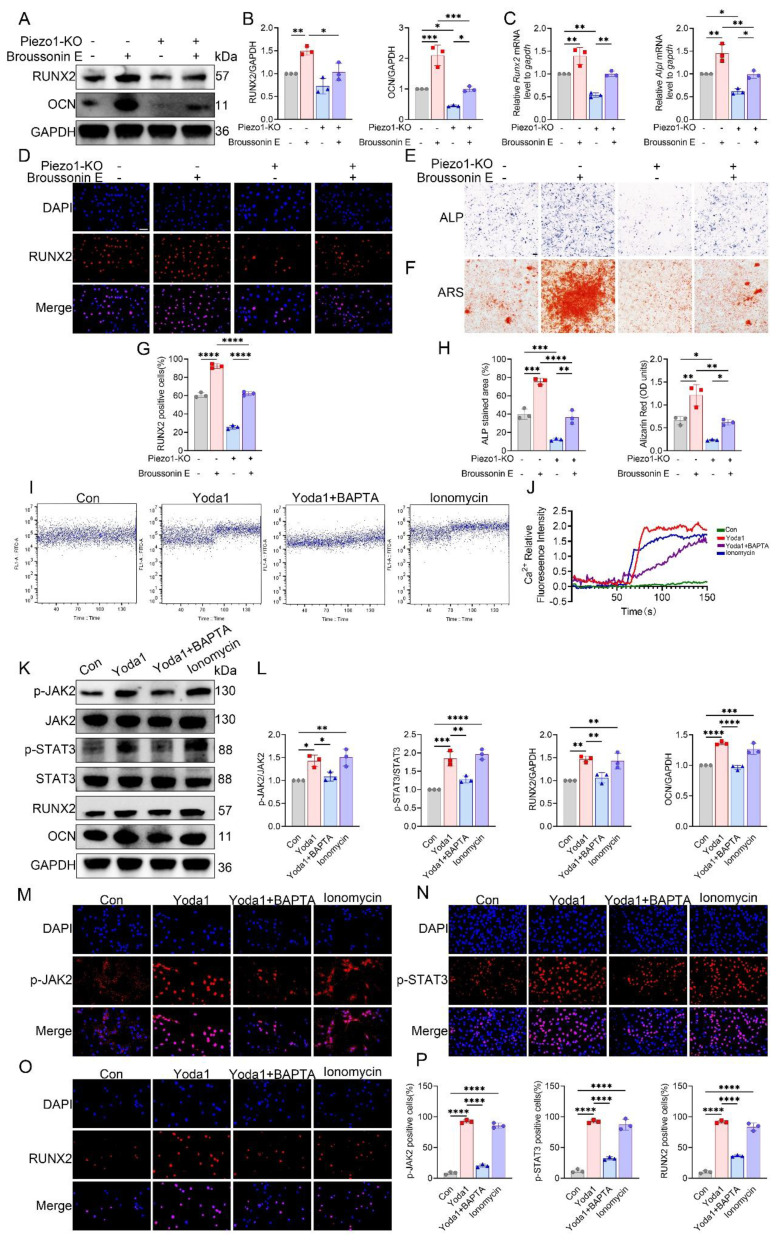
Piezo1 activates the Ca²⁺-JAK2/STAT3 signaling axis to promote osteogenic differentiation. (A) Western blot analysis of RUNX2 and OCN protein expression in control and Piezo1-KO MC3T3-E1 cells with or without Broussonin E (n = 3 per group). GAPDH was used as the loading control. (B) Quantification of RUNX2 and OCN protein levels normalized to GAPDH (n = 3 per group). (C) Relative mRNA expression of *Runx2* and *Alpl* detected by qPCR (n = 3 per group). (D) Representative immunofluorescence staining of RUNX2 and DAPI with or without Broussonin E (scale bar = 50 μm). (E) Representative ALP staining under the indicated conditions (scale bar = 500 μm). (F) Representative ARS staining under the indicated conditions (scale bar = 500 μm). (G) Quantification of RUNX2-positive cells based on immunofluorescence staining (n = 3 per group). (H) Quantification of ALP-stained area (%) and Alizarin Red (OD units) (n = 3 per group). (I) Representative intracellular Ca²⁺ influx traces in cells treated with Con, Yoda1, Yoda1 + BAPTA, or ionomycin. (J) Quantitative analysis of relative intracellular Ca²⁺ fluorescence intensity over time under the indicated treatments (n = 3 per group). (K) Western blot analysis of p-JAK2, total JAK2, p-STAT3, total STAT3, RUNX2, and OCN in cells treated with Con, Yoda1, Yoda1 + BAPTA, or ionomycin (n = 3 per group). (L) Quantification of p-JAK2 relative to total JAK2, p-STAT3 relative to total STAT3, and RUNX2 and OCN relative to GAPDH (n = 3 per group). (M) Representative immunofluorescence staining of p-JAK2 and DAPI under the indicated treatments (scale bar = 50 μm). (N) Representative immunofluorescence staining of p-STAT3 and DAPI under the indicated treatments (scale bar = 50 μm). (O) Representative immunofluorescence staining of RUNX2 and DAPI under the indicated treatments (scale bar = 50 μm). (P) Quantification of p-JAK2-positive, p-STAT3-positive, and RUNX2-positive cells (n = 3 per group). *p < 0.05; **p < 0.01; ***p < 0.001; **** p < 0.0001.

To further establish whether JAK2 acts downstream of Piezo1, rescue experiments were performed using the JAK2 activator Broussonin E. Western blotting and qPCR revealed that Piezo1-KO MC3T3-E1 cells showed reduced RUNX2, OCN, and ALP expression, which were substantially restored following Broussonin E treatment (Fig. 7A-C). Immunofluorescence further confirmed that Broussonin E reinstated nuclear localization of RUNX2 in Piezo1-deficient cells (Fig. 7D). Quantitative analysis supported these findings, showing a significant increase in RUNX2-positive cells upon Broussonin E administration (Fig. 7G). In parallel, ALP and ARS staining indicated that osteogenic differentiation and mineral deposition were impaired in Piezo1-KO cells, but these defects were effectively rescued by Broussonin E (Fig. 7E, F). Quantitative measurements confirmed larger ALP-positive areas and higher Alizarin Red OD values after Broussonin E treatment (Fig. 7H). Taken together, these results show that pharmacologic activation of JAK2 partially restored the reduced osteogenic readouts in Piezo1-KO cells, supporting the notion that JAK2/STAT3 acts downstream of Piezo1 in regulating osteogenic responses.

### Piezo1 mediated calcium influx activates JAK2/STAT3 signaling

To determine whether Ca²⁺ entry mediated by Piezo1 triggers JAK2/STAT3 activation, we first monitored intracellular Ca²⁺ concentrations using flow cytometry. In control MC3T3-E1 cells, both Yoda1 and ionomycin markedly elevated intracellular Ca²⁺, whereas co-treatment with the Ca²⁺ chelator BAPTA completely abolished the Yoda1-induced Ca²⁺ influx (Fig. 7I, J). In contrast, Piezo1-KO cells displayed a lower basal Ca²⁺ signal and did not show further Ca²⁺ elevation in response to Yoda1 (Fig. S7). In MC3T3-E1 cells, Western blot analysis further showed that Yoda1 and ionomycin promoted JAK2 and STAT3 phosphorylation, along with increased expression of RUNX2 and OCN, whereas the combined application of Yoda1 and BAPTA eliminated these responses without altering total protein levels (Fig. 7K, L).

Consistently, immunofluorescence staining confirmed enhanced expression of p-JAK2 and increased nuclear accumulation of p-STAT3 and RUNX2 following Piezo1 activation, which was suppressed by calcium chelation (Fig. 7M-P). Together, these findings indicate that Piezo1-induced Ca²⁺ influx activates the JAK2/STAT3 pathway, thereby mediating osteoblast mechanotransduction.

## Discussion

In this study, we identified progressive medial tibial plateau settlement in DMM-induced KOA mice, accompanied by medial subchondral bone sclerosis. This finding is consistent with clinical imaging observations that uneven tibial plateau settlement correlates with radiographic KOA severity (K-L grade) 8, 10. Although tibial plateau deformation is increasingly recognized in imaging studies, its underlying biological basis remains insufficiently defined. We therefore examined time-dependent subchondral bone remodeling and explored mechanisms of mechanotransduction that may drive this maladaptive structural change under chronic mechanical overload.

Subchondral bone serves as a critical mechanical buffer in the knee, bearing ~30% of joint load, whereas cartilage accounts for only 1-3% 39. In our longitudinal micro-CT and histological assessments, DMM-induced KOA was characterized by progressive medial subchondral sclerosis accompanied by aggravation of OA structural pathology, supporting the concept that subchondral microstructural alterations can reshape stress transfer across the osteochondral unit. Whether subchondral sclerosis represents a compensatory adaptation or a pathological process remains debated 40. Some studies propose that increased bone mass and density may enhance structural support and transiently delay OA deterioration 41-43. By contrast, others suggest that excessive remodeling reduces mechanical buffering, increases stiffness, and aggravates joint stress 22, 44, 45. Mechanistically, osteocytes in mechanically overloaded subchondral regions have been reported to increase extracellular vesicle production that can penetrate into cartilage and disturb its metabolism, thereby accelerating KOA progression 46. In parallel, high-resolution micro-CT studies of tibial plateau specimens from KOA patients described “trabecular corticalization” beneath bone-on-bone grooves, suggesting that sclerosis may reflect structural failure rather than a protective adaptation 47. Extending these observations, we show that sclerosis is not an isolated event but evolves in parallel with tibial plateau deformation: in DMM mice, medial sclerosis was accompanied by plateau widening, thinning, and an increased epiphyseal angle—features of uneven settlement—whereas the lateral compartment exhibited delayed and milder changes. Collectively, these findings support that medial sclerosis and tibial plateau settlement develop in tandem and may together exacerbate progressive mechanical imbalance.

Osteoblast activity is closely involved in bone-forming remodeling of the subchondral compartment 45, 48. In our DMM model, medial subchondral remodeling became particularly evident from 6 weeks onward and was accompanied by increased osteoblast-associated osteogenic activity. Moreover, osteoblasts derived from sclerotic subchondral regions in KOA have been reported to exhibit heightened osteogenic activity and can induce hypertrophic or mineralizing changes in chondrocytes in vitro, highlighting their active involvement in pathological joint remodeling 49-51. Consistently, a multiscale analysis of human femoral heads in symptomatic hip OA reported increased osteoblast numbers along with heterogeneous matrix mineralization as characteristic subchondral features across stages of cartilage degeneration 26. These findings encouraged us to further investigate the mechanotransduction drivers of osteoblast activation under pathological loading. Prior work has suggested that Piezo1 can participate in converting mechanical cues into Ca²⁺-linked signaling and osteogenic responses in osteoblastic cells 52-54. In line with this notion, we found that Piezo1 staining was higher in medial than lateral subchondral bone in human KOA samples, and increased in the medial subchondral region as KOA progressed in the DMM model. In vitro, mechanical stretch was accompanied by increased Piezo1 and an osteogenic response, whereas Piezo1 deficiency attenuated these load-associated changes; correspondingly, osteoblast-targeted Piezo1 deletion mitigated medial subchondral sclerosis and tibial plateau settlement. On this basis, we next explored downstream pathways potentially linking Piezo1 activation to osteogenic remodeling under overload.

The JAK2/STAT3 signaling pathway has been implicated in OA pathogenesis and bone remodeling 35, 55, 56. In cartilage, IL-6-driven STAT3 activation is associated with catabolic responses, and systemic inhibition of this pathway has been reported to alleviate structural degeneration in DMM models 57, 58. Beyond inflammation, evidence also supports a pro-osteogenic role of JAK2/STAT3; for example, in glucocorticoid-induced osteonecrosis, pathway activation promoted osteoblast differentiation and preserved trabecular microarchitecture, whereas STAT3 knockdown abolished these effects 59. Despite these advances, whether JAK2/STAT3 is engaged by mechanical overload in subchondral osteoblast-lineage cells during KOA progression has remained insufficiently defined. In our study, p-JAK2 and p-STAT3 staining were higher in medial than lateral subchondral bone in human KOA tibial plateaus, and JAK2/STAT3 activation increased progressively in the medial compartment during DMM progression, concurrent with sclerosis and uneven tibial plateau settlement. Pharmacologic inhibition with AG490 attenuated overload-associated medial subchondral remodeling. Genetic evidence further strengthened this interpretation: osteoblast-specific deletion of JAK2 alleviated subchondral sclerosis, improved tibial plateau morphology, and was accompanied by better performance in motor behavior assays, suggesting that restraining JAK2/STAT3 signaling in osteoblasts can translate structural benefits into functional improvement. At the cellular level, cyclic tensile strain induced JAK2/STAT3 phosphorylation and nuclear accumulation of STAT3, consistent with STAT3 functioning as a transcriptional effector downstream of mechanical stimulation 55, 56. Mechanistically, prior studies have linked Ca²⁺ influx to JAK2 activation through intermediates such as PYK2 60. In line with this concept, our data indicate that Piezo1-dependent Ca²⁺ entry contributes to JAK2/STAT3 activation: pharmacological inhibition of Ca²⁺ influx or Piezo1 reduced JAK2/STAT3 phosphorylation, whereas pharmacologic activation of JAK2 partially restored the reduced osteogenic readouts observed in Piezo1-deficient cells, supporting JAK2 as a downstream effector of Piezo1. Together, these results support the existence of a Piezo1-Ca²⁺-JAK2/STAT3 mechanotransduction axis linking pathological loading to osteoblast-associated subchondral sclerosis and uneven tibial plateau settlement during KOA progression, and raise the possibility that selective modulation of this axis may help temper maladaptive subchondral remodeling.

Consistent with our findings, Liu et al. reported that pharmacological JAK2 blockade with tyrphostin AG490 reduced cartilage degeneration and attenuated IL-6-induced pain sensitization in a murine OA model 61. Moreover, targeting the IL-6R/JAK2 axis improved joint homeostasis following DMM surgery in another study 62. A recent systematic review further summarized evidence implicating JAK2/STAT3 signaling in cartilage degeneration, subchondral bone remodeling, and synovial inflammation in experimental OA, and reported that pharmacological or genetic attenuation of this pathway can ameliorate OA-related structural and inflammatory changes 35.

Despite these encouraging findings, the translational implications of JAK2 inhibition warrant caution. Systemic JAK blockade can interfere with cytokine signaling and immune regulation and has been associated with infection risk, hematologic alterations, and metabolic disturbances across clinical contexts. As our study used intraperitoneal administration, joint-specific pharmacokinetics and safety of AG490 were not assessed, underscoring the need for more selective or locally targeted JAK2-modulating approaches in future work 63, 64. Similarly, Stattic, a small-molecule STAT3 SH2-domain inhibitor, is largely confined to preclinical use. Its poor water solubility and limited in vitro efficacy, along with the tendency of hydrophobic STAT3 inhibitors to show suboptimal bioavailability and non-specific cytotoxicity, have so far hindered its clinical translation and prompted exploration of nano-carrier-based formulations 65. Nonetheless, the present study has limitations that should be addressed in future work. The DMM model is injury-driven and therefore cannot fully capture the multifactorial nature of human KOA, which involves aging, metabolic stress, obesity-related inflammation, and chronic mechanical overload. In addition, because human subchondral bone tissue is difficult to obtain, the number of clinical tibial plateau specimens included in this study was limited (n = 3). Future studies may incorporate comorbidity-associated KOA models, expand clinical cohorts, and evaluate the efficacy and safety of targeted JAK2-modulating strategies to provide a more clinically grounded assessment of therapeutic potential.

**Figure 8:**
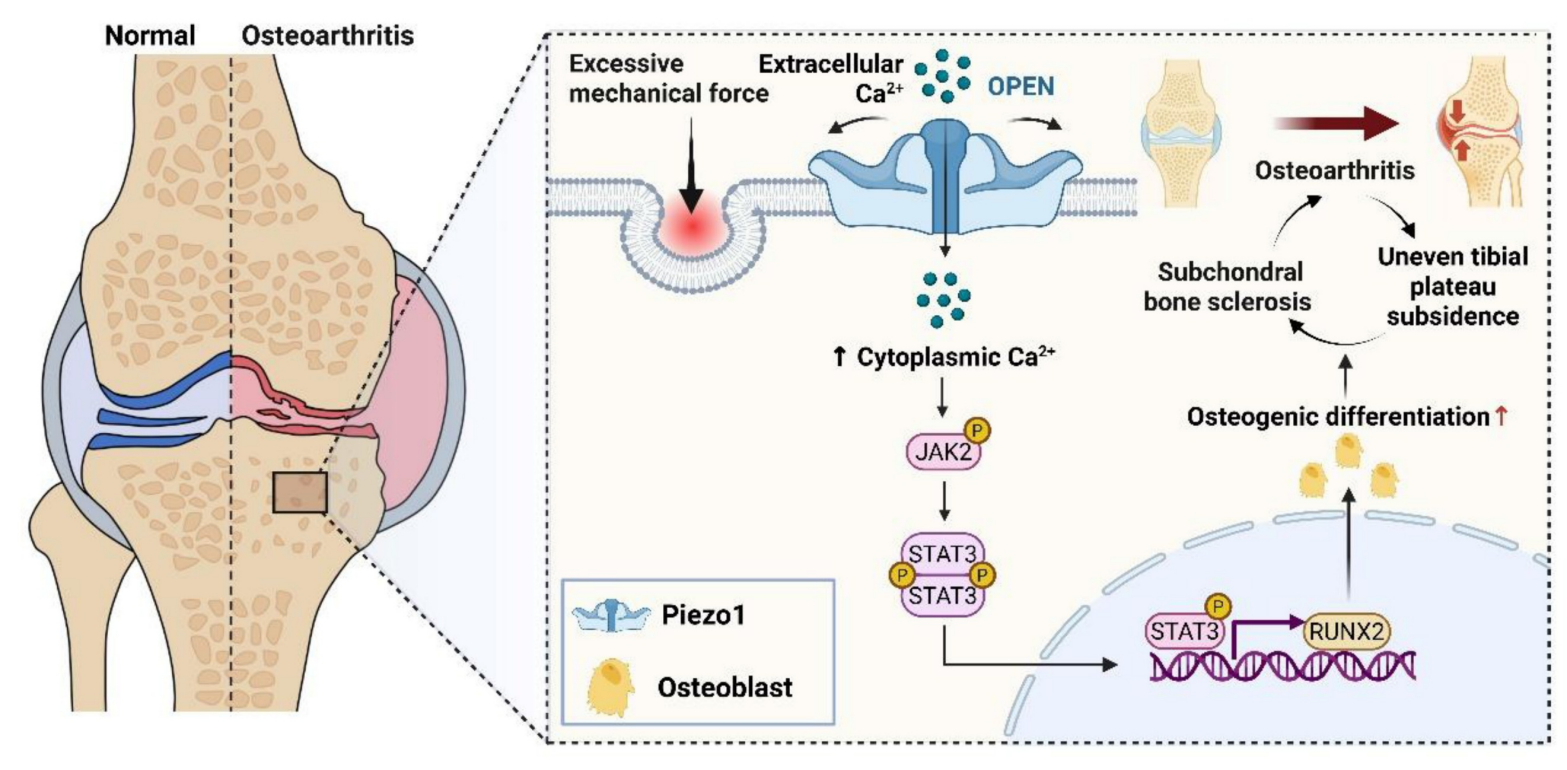
Schematic illustration showing the contribution of osteoblastic Piezo1 on osteoarthritis progression by promoting subchondral bone sclerosis via Ca²⁺ influx and JAK2/STAT3 signaling. Designed using BioRender.

In conclusion, by delineating a Piezo1-Ca²⁺-JAK2/STAT3 signaling axis in subchondral bone, our study provides mechanistic insight into how pathological mechanical stress drives sclerosis and uneven tibial plateau settlement. These findings not only advance understanding of KOA pathogenesis but also offer a rationale for developing therapies aimed at restoring subchondral bone homeostasis and correcting joint imbalance (Fig. 8).

## Supplementary Material

Supplementary figures and tables.

## Abbreviations

KOA: Knee osteoarthritis
HTO: High tibial osteotomy
DMM: Destabilization of the medial meniscus
WT: Wild-type
iCKO: inducible conditional knockout
BV/TV: Bone volume fraction
BMD: Bone mineral density
Tb.Th: Trabecular thickness
Tb.N: Trabecular number
Tb.Sp: Trabecular separation
TPEA: Tibial plateau epiphyseal angle
OCN: Osteocalcin
JAK2: Janus kinase 2
STAT3: Signal transducer and activator of transcription 3
RUNX2: Runt-related transcription factor 2
