# Supplementary material for: Abnormal mechanical load aggravates subchondral bone remodeling and uneven tibial plateau settlement in knee osteoarthritis via activation of osteoblast Piezo1-Ca²⁺-JAK2/STAT3 signaling
Source: Int J Biol Sci. 2026 Jan 15;22(4):1834–51. doi: 10.7150/ijbs.124507 (PMC12905582; doi:10.7150/ijbs.124507)
Supplement: Supplementary file 1 — Supplementary figures and tables. [file ijbsv22p1834s1.pdf]

A

MC3T3-E1：TGCGTAGCCGCCACCAGCT—GGCTATGCTCTGCTCGCCCTGCATCCTGCTGTATG—GGCTGACGCTCTGCTGCCTGAGGTACGT

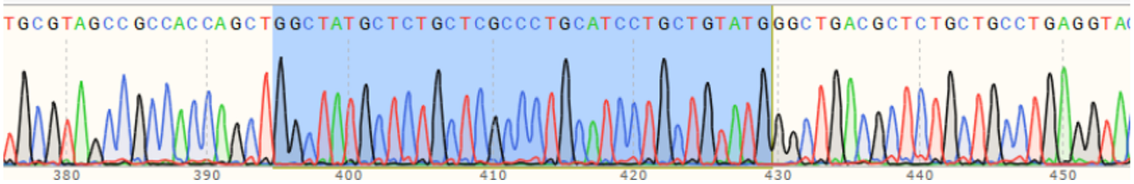

B

Piezo1-KO (Δ35 bp)：GGGCCTGCCTCATCTGGACGGTGCGTAGCCGCCACCAGCT--del 35bp--GGCTGACGCTCTGCTGCCTGAGGTACGTGTGGGCCATGGA

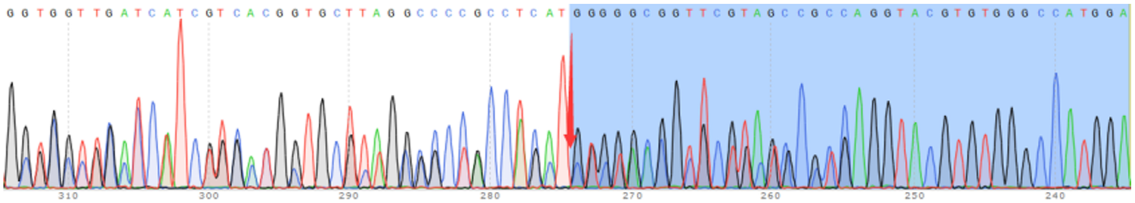

C

MC3T3-E1：TGCGTAGCCGCC—ACCAGCTGGCTATGCTCTGCTCGCCCTGCATCCTGCTGTATGGGCTGACGCTCTGCTGCCTG—AGGTACGTGTGG

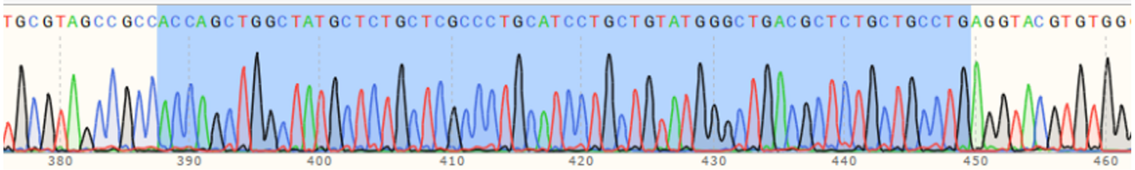

D

Piezo1-KO (Δ62 bp)：CTGCTCTGGGCCTGCCTCATCTGGACGGTGCGTAGCCGCC--del 62bp--AGGTACGTGTGGGCCATGGAACTTCCTGAGCTGCCCACCA

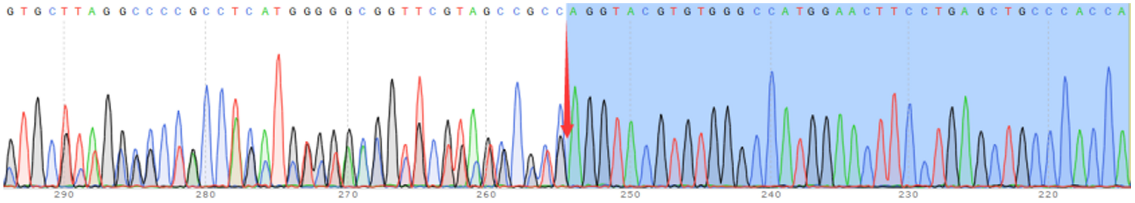

Fig. S1. (A) Chromatogram of unmodified MC3T3-E1 cells showing the wild-type sequence spanning the Δ35-bp region. (B) Chromatogram of the Piezo1-KO clone showing the annotated Δ35-bp deletion allele at the sgRNA cut site. (C) Chromatogram of unmodified MC3T3-E1 cells showing the wild-type sequence spanning the Δ62-bp region. (D) Chromatogram of the Piezo1-KO clone showing the annotated Δ62-bp deletion allele at the sgRNA cut site. Red arrows indicate the cut site; blue shading indicates mixed/out-of-phase peaks. Sequences above show the annotated Δ35-bp and Δ62-bp alleles.

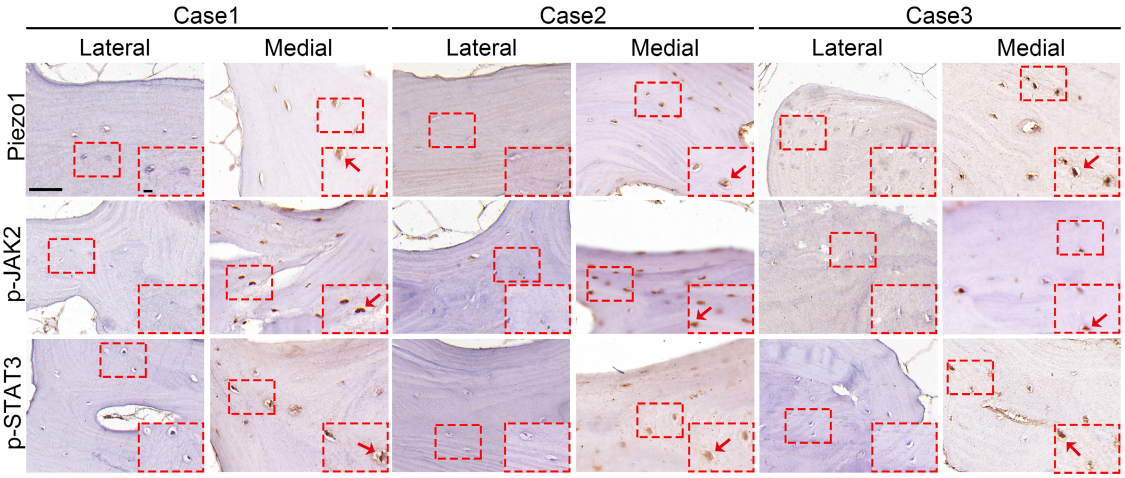

Fig. S2. Piezo1–JAK2/STAT3 signaling is upregulated in human KOA subchondral bone. Representative immunohistochemical staining for Piezo1, p-JAK2, and p-STAT3 in subchondral bone from the lateral and medial tibial plateau compartments of three patients with KOA (Case 1–3) undergoing total knee arthroplasty; red boxes indicate areas shown at higher magnification, and red arrows highlight representative positive cells (scale bar = 50 μm; enlarged views, 10 μm).

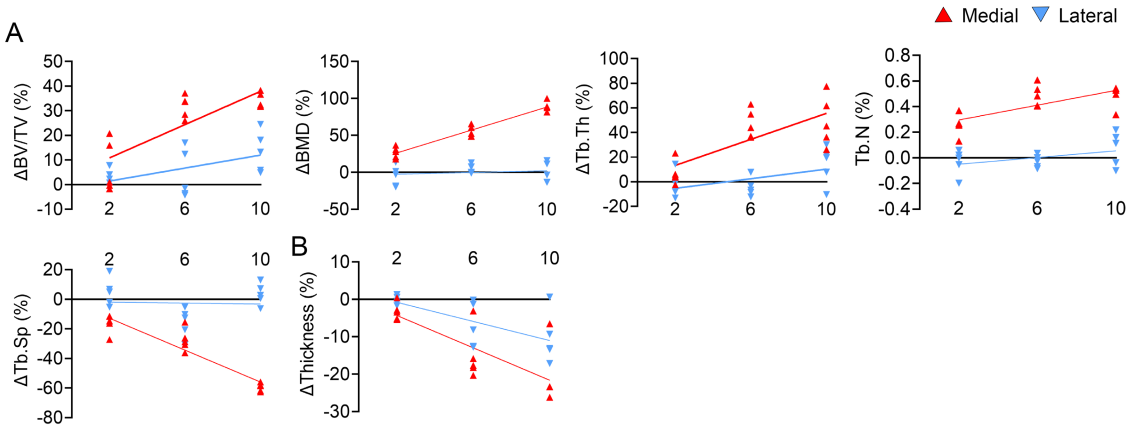

Fig. S3. Changes in medial and lateral subchondral bone microstructure and the degree of uneven settlement of the tibial plateau during DMM-induced KOA progression. (A) Quantification of relative changes (Δ%) in medial and lateral tibial plateau microstructural parameters BV/TV, BMD, Tb.Th, Tb.N, and Tb.Sp during KOA progression; (B) Quantification of relative changes (Δ%) in medial and lateral tibial plateau thickness. Data are presented as mean ± SD (n = 5 mice per group). *$p < 0.05$; **$p < 0.01$; ***$p < 0.001$; **** $p < 0.0001$.

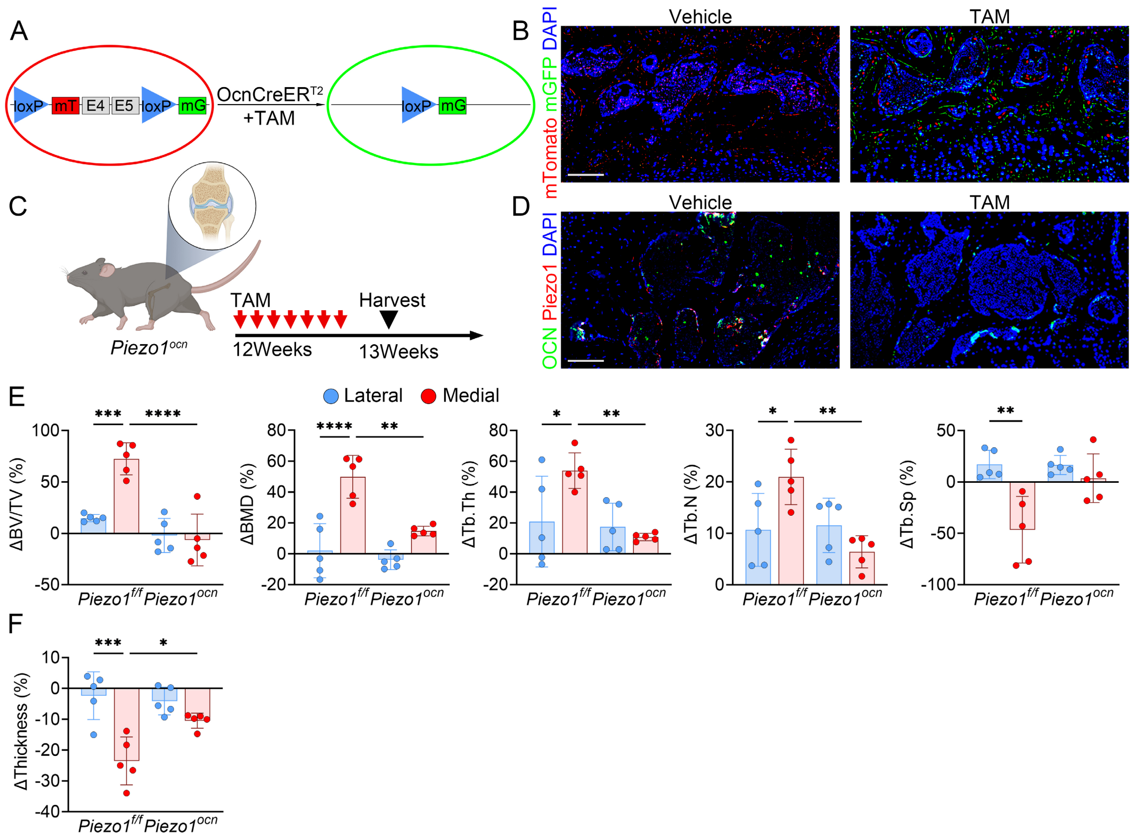

Fig. S4. Osteoblast-specific Piezo1 knockout via *OcnCreER*^{T2} alleviates settlement of the medial tibial plateau. (A) Schematic representation of the *mTmG* reporter strategy to verify *CreER*^{T2}-mediated recombination efficiency, in which TAM administration switches fluorescence from tdTomato (red) to GFP (green); (B) Representative fluorescence images of tibial subchondral bone showing reporter signal before Vehicle and after TAM induction, confirming *CreER*^{T2} activation (scale bar = 100 μm); (C) Schematic of the TAM induced osteoblast-specific Piezo1 knockout strategy in 12 week old *Piezo1*$^{f/f}$ and *Piezo1*^{ocn} mice; (D) Representative immunofluorescence staining of OCN (green) and Piezo1 (red) in tibial subchondral bone before and after TAM treatment, validating efficient Piezo1 deletion (scale bar = 100 μm); (E) Quantification of relative changes (Δ%) in medial and lateral tibial plateau bone structural parameters BV/TV, BMD, Tb.Th, Tb.N, and Tb.Sp in *Piezo1*$^{f/f}$ and *Piezo1*^{ocn} mice under DMM conditions (n = 5 per group); (F) Quantification of relative changes (Δ%) in medial and lateral tibial plateau thickness between genotypes (n = 5 mice per group). *$p < 0.05$; **$p < 0.01$; ***$p < 0.001$; **** $p < 0.0001$.

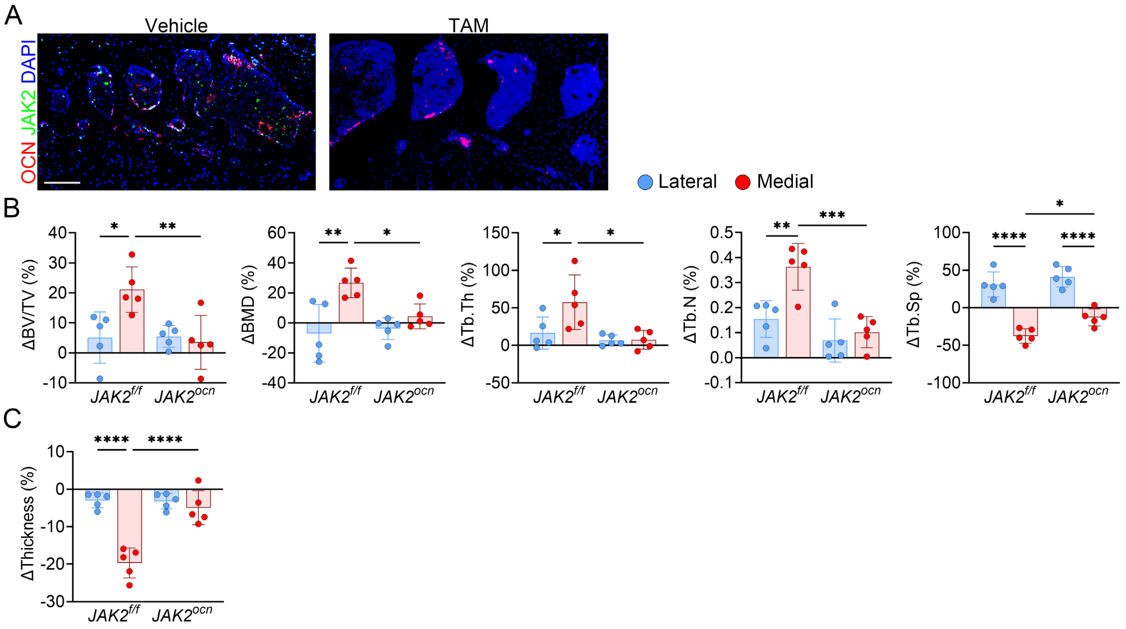

Fig. S5. Osteoblast-specific deletion of JAK2 under DMM conditions alleviates subchondral bone sclerosis and settlement of the medial tibial plateau. (A) Representative immunofluorescence staining showing the expression of OCN (red) and JAK2 (green) in the tibial subchondral bone of Vehicle and TAM mice (scale bar = 100 μm); (B) Quantification of relative changes (Δ%) in medial and lateral tibial plateau bone structural parameters BV/TV, BMD, Tb.Th, Tb.N, and Tb.Sp in *JAK2$^{f/f}$* and *JAK2^{ocn}* mice under DMM conditions (n = 5 per group); (C) Quantification of relative changes (Δ%) in medial and lateral tibial plateau thickness between genotypes (n = 5 mice per group). *p < 0.05; **p < 0.01; ***p < 0.001; **** p < 0.0001.

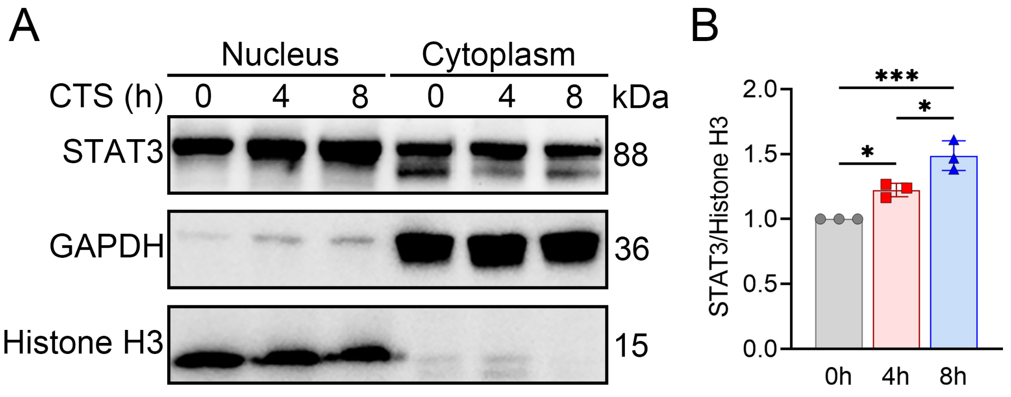

Fig. S6. Mechanical stimulation promotes nuclear accumulation of STAT3 in osteoblasts. (A) Western blot analysis of STAT3 in nuclear and cytoplasmic protein fractions of MC3T3-E1 cells subjected to 10% cyclic tensile strain (CTS, 0.5 Hz) for 0, 4, or 8 h. Histone H3 and GAPDH served as nuclear and cytoplasmic loading controls, respectively; (B) Quantification of nuclear STAT3 levels normalized to Histone H3 at 0, 4, and 8 h after CTS. *$p < 0.05$; **$p < 0.01$; ***$p < 0.001$.

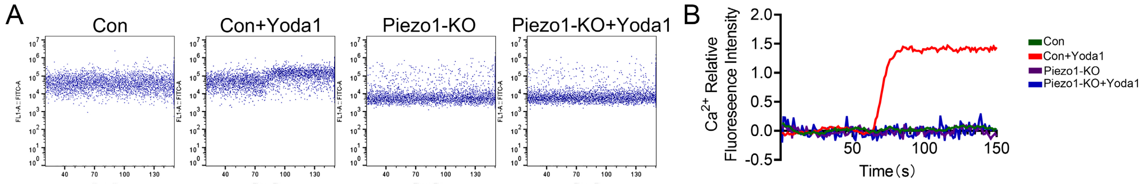

Fig. S7. Yoda1-induced Ca^{2+} influx is abolished in Piezo1-KO osteoblasts. (A) Representative intracellular Ca^{2+} influx traces in control MC3T3-E1 cells and Piezo1-KO MC3T3-E1 cells treated with vehicle or Yoda1. Yoda1 markedly increased intracellular Ca^{2+} levels in control cells but not in Piezo1-KO cells; (B) Quantitative analysis of relative intracellular Ca^{2+} fluorescence intensity over time under the indicated treatments (n = 3 per group).

Table S1. Genotyping primers used for conditional knockout mouse lines

Gene	Primer	Sequence (5'-3')	Type
$JAK2^{f/f}$	Forward	ATCCAGTGGCTTGTTATTATCTCA	Forward
	Reverse	TCAGCAGAAACTCCAGTCGC	Reverse
$Piezo1^{f/f}$	Forward	GCCTAGATTCACCTGGCTTC	Forward
	Reverse	GCTTTAACCATTGAGCCATCT	Reverse
$OcnCreER^{T2}$	P1	GAGGGTGTGTCTATCCCGTG	Forward
	P2	CCCCTATCACCACCACGATG	Forward
	P3	CCTCCTCATCCTCTCCCACA	Reverse
	P4	AGCAGCGTATCCACATAGCG	Reverse
$Rosa26^{mTmG}$	P1	TAGAGCTTGCGGAACCCTTC	Forward

	P2	CTTTAAGCCTGCCCAGAAGA	Reverse
	P3	AGGGAGCTGCAGTGGAGTAG	Forward

Table S2. PCR and sequencing primers used to confirm Piezo1 knockout in MC3T3-E1 cells

Forward primer (5′-3′)	Reverse primers (5′-3′)	Sequencing primer (5′–3′)
TGTGCCCTGATTGCCATGAT	CATACTCGGTCACAACGCCT	CATACTCGGTCACAACGCCT
